# Integrated transcriptomic and physiological analysis reveals cadmium stress responses in kiwifruit rootstock *Actinidia valvata* via an optimized *Agrobacterium rhizogenes*-mediated hairy root transformation system

**DOI:** 10.3389/fpls.2026.1818881

**Published:** 2026-04-28

**Authors:** Miaofeng Gu, Junjie Zhu, Junyao Xia, Yibing Zeng, Yongbin Gao, Liuqing Huo, Zhi Xu, Jinhua Liu, Xuepeng Sun, Kai Xu, Haijie Ma

**Affiliations:** 1 Key Laboratory of Quality and Safety Control for Subtropical Fruit and Vegetable, Ministry of Agriculture and Rural Affairs, Collaborative Innovation Center for Efficient and Green Production of Agriculture in Mountainous Areas of Zhejiang Province, College of Horticulture Science, Zhejiang A&F University, Hangzhou, Zhejiang, China; 2 Analysis and Test Center, Hainan Provincial Key Laboratory of Quality and Safety for Tropical Fruits and Vegetables, Key Laboratory of Quality and Safety Control for Subtropical Fruit and Vegetable, Key Laboratory of Tropical Fruits and Vegetables Quality and Safety for State Market Regulation, Chinese Academy of Tropical Agricultural Sciences, Haikou, China; 3 Yangsheng Tang Co., Ltd. No 181, Geyazhuang, Xihu District Hangzhou, Zhejiang, China

**Keywords:** *A. valvata*, Cd^2+^, genetic transformation, kiwifruit, rootstock, transcriptomics

## Abstract

**Abstract:** Kiwifruit (*Actinidia* spp.) rootstocks play a crucial role in enhancing environmental adaptability, yet the lack of efficient genetic transformation systems has limited functional genomic studies on stress resistance mechanisms. Here, we established a rapid and cost-effective *Agrobacterium rhizogenes*-mediated hairy root transformation system for the widely used rootstock *Actinidia valvata*, bypassing the need for tissue culture. This system achieved transgenic root production within 3–5 weeks and demonstrated broad applicability in both *Myrica rubra* and *Actinidia chinensis*. Utilizing GFP as a viability marker, we observed that 25 µM Cd²^+^ markedly impaired root cell physiology within 2 h, preceding visible phenotypic changes. Transcriptomic profiling of Cd²^+^-treated roots identified extensive differential gene expression, with enrichment in key pathways such as “Plant hormone signal transduction” and “MAPK signaling pathway.” Hormonal quantification further revealed significant alterations in IAA, IBA, ABA, JA, SA, and GA levels under Cd²^+^ stress. Our integrative multi-omics approach, combining transcriptomics, hormone profiling, and physiological validation, provides a comprehensive framework for decoding genotype-to-phenotype relationships in woody horticultural species. The established transformation platform not only enables efficient functional gene verification in kiwifruit roots but also provides a reliable platform for dissecting root-specific cadmium stress responses in Ericales woody species.

## 1 Introduction

Kiwifruit (*Actinidia* spp.), a deciduous perennial vine originating from China, has gained global commercial significance due to its distinctive flavor profile and high nutritional value, particularly its rich content of vitamin C, polyphenols, and dietary fiber (Huang et al., 2013). The genus *Actinidia* comprises more than 50 species (Huang and Ferguson, 2007), with worldwide production exceeding 4 million tons in 2023 (FAOSTAT). Furthermore, the absence of efficient genetic transformation systems has impeded functional characterization of stress-responsive genes and molecular mechanisms underlying rootstock performance. Current evaluation methods rely predominantly on phenotypic and physiological assessments, which are labor-intensive and often fail to reveal underlying genetic and regulatory pathways. Thus, the development of a highly efficient and genotype-independent transformation platform for kiwifruit rootstocks is urgently needed to advance functional genomics and precision breeding. However, the lack of an efficient hairy root transformation system limits the research on cadmium stress response mechanisms in kiwifruit rootstocks.

Currently, the establishment of efficient and reproducible genetic transformation systems remains a major bottleneck for functional genomics research in many perennial crops, including kiwifruit. *Agrobacterium* species, including *Agrobacterium tumefaciens* and *Agrobacterium rhizogenes*, are commonly used for genetic transformation (Krenek et al., 2015; Bahramnejad et al., 2019; Goralogia et al., 2025). Although *A. tumefaciens*-mediated transformation has been achieved in a limited number of *Actinidia* genotypes (e.g., *Actinidia chinensis*), its utility is constrained by characteristically low transformation efficiency, pronounced genotype dependence, and extended regeneration timelines, limitations commonly observed across many fruit tree species (Nagle et al., 2018; Varkonyi-Gasic et al., 2019; Wang et al., 2022; Fu et al., 2023; Chen et al., 2024). These limitations are especially evident in rootstock species, where conventional methods often fail to directly and efficiently generate transgenic roots, the key organs involved in stress response. In contrast, *A. rhizogenes*-mediated genetic transformation generally offers higher efficiency than *A. tumefaciens*, and is widely applicable to various plants, including non-model herbaceous species and woody plants (Ma et al., 2022; Liu et al., 2023; Ma et al., 2023; Ramasamy et al., 2023; Wang et al., 2025; Yin et al., 2025). This system allows the rapid production of composite plants (consisting of transgenic roots and wild-type shoots), which bypasses the requirement for whole-plant regeneration and proves particularly advantageous for investigating root biology and root-environment interactions directly in rootstock species (Meng et al., 2019; Cao et al., 2023). Notably, recent advancements have demonstrated the feasibility of bypassing tissue culture through *in planta* transformation strategies in citrus (Zhang et al., 2025), substantially shortening experimental timelines and reducing labor requirements. Given the high cutting survival rate and robust root regeneration capacity of *A. valvata*, it serves as an ideal model for developing such an advanced transformation system in kiwifruit rootstocks. *A. rhizogenes*-mediated hairy root transformation is the optimal method for root-specific functional studies, which is perfectly suitable for analyzing root responses to cadmium stress in kiwifruit rootstocks.

Rootstocks primarily exist underground as roots, making them highly susceptible to adverse environmental factors in the soil, such as heavy metal (HM) stress. HMs encompass 52 elements, including Cd, Pb, Mn, Cu, Ni, Co, Hg, and As, which impact plant performance in a concentration-dependent manner (Nagajyoti et al., 2010; Boyd and Rajakaruna, 2013). Although certain HMs (e.g., Zn) are essential for plant growth, most are toxic (Broadley et al., 2007; Zhang and Song, 2018). Once absorbed by plant roots, heavy metals accumulate in various tissues, severely disrupting physiological and molecular processes (Seregin et al., 2004; Rai et al., 2016). Cadmium (Cd) is a highly toxic, non-essential heavy metal widely present in the environment and is among the three most hazardous contaminants, alongside Hg and Pd, posing significant risks even at low concentrations (Jamers et al., 2013; Ismael et al., 2019; Mushtaq et al., 2025). Excessive Cd accumulation disrupts physiological processes in plants, reducing chlorophyll and carotenoid content, impairing photosynthesis and biomass, and causing protein dysfunction (Asgher et al., 2015; ur Rehman et al., 2018; Huihui et al., 2020; Imran et al., 2020; Hussain et al., 2021; Riaz et al., 2021; Soni et al., 2024; Zhu et al., 2021). Cd contaminates the environment through sources such as metalworking industries, urban composting, phosphate-based fertilizers, sewage irrigation, rock weathering, and forest fires (Masindi and Muedi, 2018; Shanmugaraj et al., 2019; Augustsson et al., 2018). Cd primarily exists in soil as the divalent cation Cd²^+^, and its absorption by roots is enhanced by the negative gradient generated by the plasma membrane (Li et al., 2023). This uptake is mediated by transporters or channels developed for essential metals like Cu, Fe, Zn, and Mn (Llugany et al., 2012; Ur Rahman et al., 2021; Song et al., 2017; Ni et al., 2020). The cellular uptake and internal translocation of Cd²^+^ are primarily mediated by members of the NRAMP, ZIP, and YSL transporter families (Huang et al., 2020). Roots primarily supply water and nutrients to the plant, with root hairs playing a key role in absorbing Cd²^+^ from the soil. They enhance root penetration and ion uptake, even in low-diffusion conditions, with root hair density in *Arabidopsis thaliana* positively correlating with Cd exposure (Brown et al., 2017; Kohanová et al., 2018). Kiwifruit is highly sensitive to Cd, but the potential mechanisms of its impacts remain poorly understood. With their extensive root systems, kiwifruit rootstocks readily absorb soil Cd, making them particularly vulnerable to its toxicity. Therefore, understanding the effects of Cd on kiwifruit is essential for ensuring safe and sustainable production.

This study aims to establish a genetic transformation system for kiwifruit rootstocks and use it to investigate the physiological and molecular effects of Cd on root cells. By utilizing *A. rhizogenes*’ ability to induce transgenic hairy roots in many plant species and the high survival rate of kiwifruit rootstock cuttings, we developed an efficient genetic transformation system in *A. valvata* bypassing tissue culture. Using this method, we generated GFP-overexpressing transgenic hairy roots and conducted utilized GFP fluorescence as an *in vivo* marker for the rapid evaluation of cell viability under various Cd concentrations. The study also analyzed the impact of Cd treatment on gene transcription and hormone content in the kiwifruit root system. Notably, we demonstrated that the established transformation system is also effective for the more difficult-to-transform species, *M. rubra*. Furthermore, we established a simple and efficient method for inducing shoots from transgenic root in kiwifruit. In summary, we developed a versatile hairy root transformation system for woody species in the Ericales (kiwifruit and bayberry) and applied it to investigate plant cell responses to environmental stress. Future work will test its applicability to other fruit tree families.

## 2 Materials and methods

### 2.1 Plant materials and growth conditions

All 1-year-old semi-lignified branches of kiwifruit and bayberry were collected from clonally propagated mature vines. The tested materials included *A. valvata* Dunn (‘DE’), *A. chinensis* ‘Hongyang’ (‘HY’), *A. chinensis* ‘Donghong’ (‘DH’), *A. chinensis* ‘Xuxiang’ (‘XX’), and *M. rubra* cv. ‘Dongkui’. All source vines were maintained under open-air natural conditions at the Kiwifruit Germplasm Resource Nursery of the College of Horticulture Science, Zhejiang A&F University. The subsequent cuttings and plants were cultivated in a greenhouse at 26°C with a 16h/8h (light/dark) photoperiod. Both the solid cutting and liquid hydroponic treatments were strictly maintained under identical environmental conditions to ensure comparability.

### 2.2 Bacterial strains and plasmids

The competent *A. rhizogenes* cells of K599 (CAT#: AC1082) and ATCC15834 (CAT#: AE1100), as well as competent *Escherichia coli* DH5α cells (CAT#: DL1001), were purchased from Shanghai Weidi Biotechnology Co., Ltd. The pathogen of kiwifruit bacterial canker (*Pseudomonas syringae* pv. *Actinidiae*, *Psa*) used in this study is maintained in our lab. The cultivation conditions for K599 and ATCC15834 were TY medium at 28 °C; for DH5α, LB medium at 37 °C; and for *Psa*, LB medium at 25 °C. All bacterial strains were stored in 50% glycerol and kept at -80 °C. The activated *A. rhizogenes* strains and *E. coli* cells containing the target plasmids were screened and cultured using Kanamycin (50 μg/mL) and Streptomycin (50 μg/mL) depending on the vector and strain resistance.

Binary vectors including GFP-ER (endoplasmic reticulum marker, **Supplementary Table 1**), pDSK-GFPuv (for overexpressing *GFP* in *Psa*, **Supplementary Table 1**), GFP-PX (peroxisomes marker, http://nebenfuehrlab.utk.edu/markers/default.htm), GFP-Lifeact (actin marker) (Gong et al., 2024), were used in this study.

### 2.3 Reagents and medium

MES infiltration solution: 10 mM MgCl_2_, 10 mM MES [2-(N-morpholino) ethanesulfonic acid, CAS: 4432-31-9], 100 μM acetosyringone, pH=5.2.
CTAB extraction solution: 4 g CTAB, 16.364 g NaCl, 20 mL 1 M Tris-HCl (pH=8.0), 8 mL 0.5 M EDTA, add H_2_O to 200 mL.
LB solid medium: 5 g/L Yeast extract, 10 g/L Tryptone, 10 g/L NaCl, 15g/L Agar, pH=7.0.
LB liquid medium: 5 g/L Yeast extract, 10 g/L Tryptone, 10 g/L NaCl, pH=7.0.
TY solid medium: 3 g/L Yeast extract, 5g/L Tryptone, 10 mM CaCl_2_, 15g/L Agar, pH=7.0.
TY liquid medium: 3 g/L Yeast extract, 5g/L Tryptone, 10 mM CaCl_2_, pH=7.0.
MS basal medium: 4.43 g/L MS, 30 g/L Sucrose, 8 g/L Agar, pH=5.8.
Callus and shoot induction medium: MS basal medium supplemented with 400mg/L cef (cefotaxime) and varying concentration of 6-BA and NAA.
Co-culture medium: MS basal medium supplemented with 1 mg/L TDZ, 0.5 mg/L NAA, 0.5 mg/L Bet (Betaine CAS: 107-43-7), 100 μM AS (acetosyringone).
Root induction medium: MS basal medium supplemented with 400mg/L cef, 0.6 mg/L IBA.
Acetosyringone (CAS: 2478-38-8) stock solution: Dissolve acetosyringone in DMSO to a concentration of 50 mg/mL. Filter the solution through a bacterial filter to remove any bacteria, and store at -20°C.
Stock solution of 6-BA (6-Benzylamino purine, CAS: 1214-39-7), NAA (1-naphthaleneacetic acid, CAS: 86-87-3), IBA (3-Indole butyric acid, CAS: 133-32-4), and 2,4-D (2,4-Dichlorophenoxy acetic acid, CAS: 94-75-7): Dissolve in a small amount of 1 M NaOH, then dilute with sterile water in a volumetric flask to a final concentration of 2 mg/mL. Filter through a sterile filter to remove bacteria and store at -20 °C.
IAA (3-Indole acetic acid, CAS: 87-51-4) stock solution: Dissolve in a small amount of 95% ethanol, then dilute with sterile water in a volumetric flask to a final concentration of 1 mg/mL. Filter through a sterile filter to remove bacteria and store at -20 °C.
ZT (Zeatin, CAS: 13114-27-7) stock solution: Dissolve ZT in DMSO to a concentration of 1 mg/mL. Filter the solution through a bacterial filter to remove any bacteria, and store at -20°C.
Stock solution of azoxystrobin (CAS: 143390-89-0) and chlorothalonil (CAS: 1897-45-6): Dissolve azoxystrobin and chlorothalonil in DMSO to a concentration of 10000 ppm. Filter the solution through a bacterial filter to remove any bacteria, and store at -20°C.

### 2.4 *A. rhizogenes* mediated genetic transformation

Preparation of *Agrobacterium* infiltration solution: The *A. rhizogenes* strains containing the target plasmid were activated by culturing on TY medium supplemented with the corresponding antibiotics at 28 °C for 2 days. Single colonies were picked and transferred to 1 mL of TY liquid medium (with multiple replicates) and incubated overnight at 28 °C with shaking at 200 rpm. After PCR verification of the bacterial culture, the bacterial suspension was transferred to fresh TY liquid medium at a 1:20 volume ratio and cultured overnight. The bacterial culture was then centrifuged (6000 rpm, 10 minutes) to collect the cells. The collected cells were resuspended in infiltration solution and adjusted to an OD_600_ of 0.7-0.8, then incubated in the dark at room temperature for 3–5 hours before use.

*A. rhizogenes*-mediated genetic transformation: Healthy, 1-year-old semi-lignified kiwifruit branches were selected and trimmed into 8–9 cm branches, each containing at least one bud (the bud should be located near the middle of the segment). The upper end of each segment was cut flat, while the base was angled to increase the wound surface area, facilitating better contact with the bacterial solution. The branches were placed in a beaker containing *Agrobacterium* infiltration solution, ensuring that the angled base of the branches was fully submerged. The submerged segments were transferred to a vacuum chamber and subjected to vacuum infiltration for 30 minutes. After agroinfiltration, the segments were placed in trays filled with sterilized vermiculite (2–3 mm particle size), which was moistened. The trays were covered with a transparent dome to maintain optimal moisture conditions for rooting. The trays were incubated in a light incubator at 24-26 °C with a 14h/10h (light/dark) photoperiod and a light intensity of 10,000 Lux. The trays were ventilated daily for 30 minutes to prevent pathogen growth. The genetic transformation method for *M. rubra* branches and leaves is similar to that for kiwifruit branches, with the key difference being that the light intensity during the cultivation of agroinfiltrated *M. rubra* branches and leaves is set at 2500 Lux.

Identification of Transgenic Hairy Roots: 2–3 weeks after agroinfiltration, significant callus formation can be observed at the infiltration sites of both kiwifruit branches and *M. rubra* leaves. Hairy roots begin to form 4–8 weeks after agroinfiltration. Although the binary vectors contained antibiotic resistance genes, plant selection antibiotics (such as Kanamycin) were intentionally omitted during root induction to avoid potential phytotoxicity and severe inhibition of rooting commonly observed in woody plants. Instead, rapid and non-destructive identification of transgenic hairy roots was achieved exclusively by screening for GFP fluorescence using a handheld excitation light source (SUNLONGE SL8300). In all comparative assays, the ‘wild type’ (WT) or ‘control group’ refers to hairy roots that naturally developed from cuttings. To ensure a rigorous comparison, these control cuttings were treated with an empty vector (or mock solution) under identical conditions, without the application of any exogenous rooting hormones.

### 2.5 Microscopy

Transgenic hairy roots were washed with sterile water, with wild-type roots as controls. Both cross-sections and longitudinal sections of the roots were prepared using a razor blade. A confocal laser scanning microscope (OLYMPUS FV3000) was used to image the sections, with an excitation wavelength of 488 nm and an emission range of 505–550 nm, employing 10×,20× and 40× objective lenses.

### 2.6 RNA extraction and qRT-PCR analysis

Transgenic hairy roots were specifically selected as the analysis samples because the root system serves as the primary functional interface for rootstocks directly exposed to soil-borne Cd²^+^ stress. Hairy roots were cut into 2–3 mm segments and placed into 2 mL RNase-free centrifuge tubes containing two sterilized grinding beads. The tubes were snap-frozen in liquid nitrogen, then quickly transferred to a liquid nitrogen-precooled grinding adapter. The adapter was mounted on a grinder, and the roots were ground into a fine powder with two 30-second cycles at 60 Hz. RNA extraction was performed using the FastPure Universal Plant Total RNA Isolation Kit (RC411, Vazyme). RNA concentration and quality were assessed using Nanodrop and agarose gel electrophoresis. RNA meeting quality standards was reverse-transcribed using the Evo M-MLV Reverse Transcription Premix Kit (AG11728, Accurate Biotechnology). RT-qPCR analysis was carried out with the AceQ Universal SYBR qPCR Master Mix (Q511-02, Vazyme) on a real-time PCR system (Q2000A, LongGene). Actin was used as the internal control gene, and the primer sequences are provided in **Supplementary Table 2**.

### 2.7 Transcriptome sequencing and differentially expression gene (DEG) analysis

Specifically, RNA was extracted from *A. valvata* roots treated with 50 μM Cd²^+^ for 0 h (control), 2 h, and 24 h (with three biological replicates per time point). RNA samples meeting quality standards were used for library preparation and sequenced using the BGI high-throughput sequencing platform DNBSEQ. The raw data were filtered using fastp (Chen et al., 2018) to obtain valid data. Quality control of the filtered data was performed using FastQC (Davis et al., 2021). The filtered transcriptome sequences were aligned to the reference genome using STAR (Dobin et al., 2013), and statistical analysis was conducted. RSEM (Li and Dewey, 2011) was used to determine the number of reads mapped to each transcript for each sample, followed by FPKM (Fragments Per Kilobase per Million bases) conversion. Differential expression analysis was performed using the gene expression data (read counts) obtained from the quantification of gene expression in various samples. DESeq2 software (Love et al., 2014) was used for differential expression analysis, with a selection threshold of padj < 0.05 and |log2FoldChange| > 1. GO and KEGG enrichment analyses of differentially expressed transcripts were conducted using clusterProfiler (Yu et al., 2012). Transcription factors were annotated and analyzed using PlantTFDB (Jin et al., 2017).

### 2.8 Transgenic callus induction

Transgenic and wild-type hairy roots of kiwifruit grown in vermiculite were washed with sterile water. Fine root hairs were removed, leaving thicker root segments, which were soaked in 400 mg/L cef for 12 hours. In a sterile environment, the root segments were treated sequentially: soaked in 75% ethanol for 10 s; rinsed twice in sterile water for 2 minutes to remove ethanol; immersed in 1% sodium hypochlorite for 1–2 mins; rinsed again twice in sterile water to remove sodium hypochlorite. The root segments were cut into 1 cm pieces, removing lateral rootlets and tips, then blotted dry and briefly air-dried. The root segments were placed on callus induction medium and incubated in darkness at 26 °C for one week. Afterward, the segments were transferred to a growth chamber with a 14h/10h (light/dark) photoperiod and a light intensity of 10,000 Lux. Swelling of the root segments was observed after 2 weeks, with distinct callus formation appearing by 3–4 weeks.

### 2.9 Cd²^+^ and Al³^+^ stress treatments

The rootstock *A. valvata* was cultured in a hydroponic box (38 cm × 28 cm × 14 cm) with a water level of 10 cm. The solution contained Hogland’s nutrient mix, along with specified concentrations of CdCl_2_ (CAS: 7446-70-0) and AlCl_3_ (CAS: 10108-64-2). Oxygen was continuously supplied by an ACO-002 pump. The plants were grown at 26 °C with a 14h/10h (light/dark) photoperiod. The nutrient solution was replaced daily, and phenotypic changes were photographed and recorded.

## 3 Results

### 3.1 Disease resistance, flood tolerance, and cutting survival rate analysis in kiwifruit

**Figure 1:**
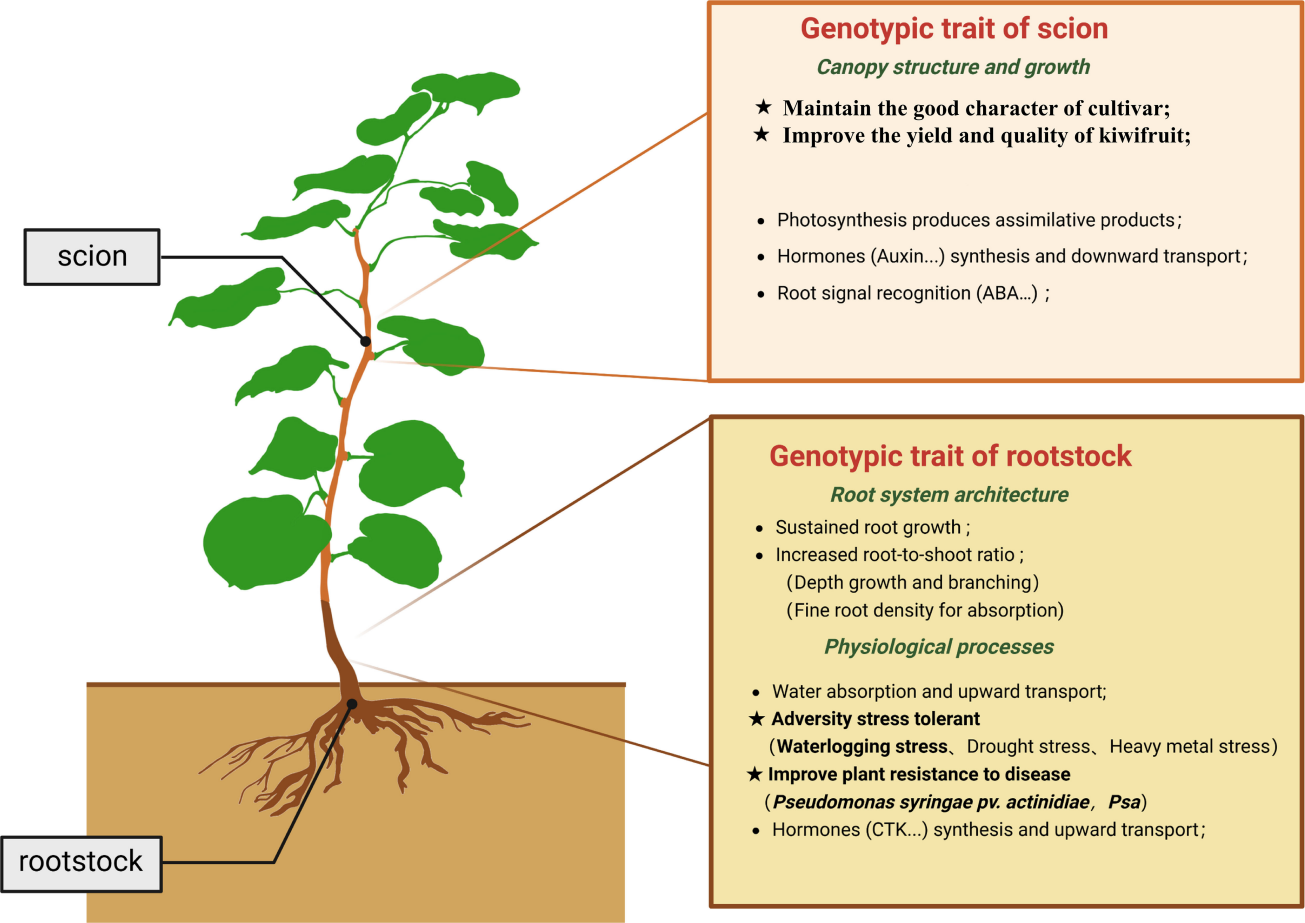
Schematic diagram of the functions of kiwifruit rootstock and scion.

**Figure 2:**
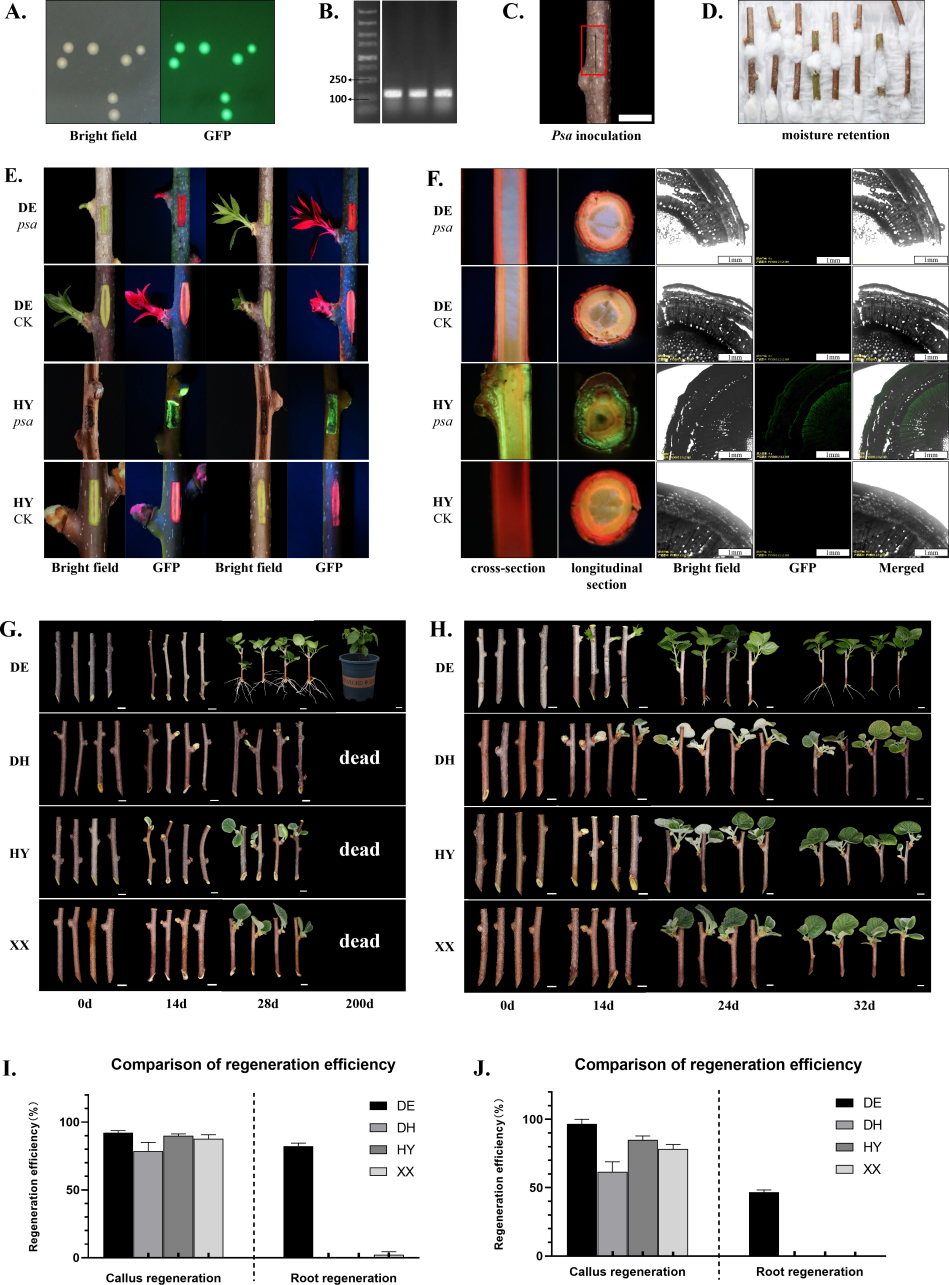
Analysis of physiological traits in kiwifruit. **(A)** Verification of *GFP* overexpressing *Psa* strain (*Psa-GFP*); **(B)** PCR verification of *GFP* sequence in *Psa-GFP*; **(C)** Schematic diagram of *Psa-GFP* inoculation in kiwifruit. **(D)** The inoculated kiwifruit branches were covered with cotton at the inoculation site to maintain moisture and placed in a humidity box. **(E)** A handheld excitation light source was used to detect *Psa-GFP* in the kiwifruit branches. Green fluorescence indicates the presence of *Psa-GFP*, while red indicates its absence. ‘DE-*psa*’ and ‘DE-CK’ represents A. valvata branches inoculated with or without *Psa-GFP*; ‘HY-*psa*’ and ‘HY-CK’ represents **(A)** chinensis ‘HY’ branches inoculated with or without *Psa-GFP*. **(F)** Both a handheld excitation light source and a confocal microscope were used to detect the presence of *Psa-GFP* in the kiwifruit branches. Green fluorescent spots indicate the existence of *Psa-GFP*. The dramatic reduction of GFP signal in ‘DE’ compared to the highly susceptible ‘HY’ visually demonstrates the robust disease resistance of *A. valvata* against kiwifruit bacterial canker. Branches of four kiwifruit species under cutting **(G)** and hydroponic **(H)** conditions. Analysis of callus formation efficiency and root formation efficiency of the four kiwifruit branches under cutting **(I)** and hydroponic **(J)** conditions. ‘DE’ represents *A. valvata* Dunn, ‘DH’ represents *A. chinensis* ‘Donghong’, ‘HY’ represents *A. chinensis* ‘Hongyang’, ‘XX’ represents *A. chinensis* ‘Xuxiang’.

The rootstock and scion are vital components of cultivated kiwifruit plants, with an ideal rootstock characterized by disease resistance, stress tolerance, and a robust root system (**Figure 1**). Since *A. valvata* is a commonly used rootstock in kiwifruit production, we conducted a comprehensive analysis of its disease resistance, flood tolerance, and cutting survival rate. Kiwifruit bacterial canker, a significant challenge in kiwifruit cultivation, can lead to plant death. However, the disease develops slowly after inoculation, making early symptoms difficult to observe and significantly hindering research efficiency. To facilitate the observation of *Psa* infection in kiwifruit tissues, the plasmid pDSK-GFPuv (**Supplementary Table 1**) was introduced into *Psa* via heat shock transformation method. The resulting GFP-overexpressing strain (*Psa*-GFP) emits green fluorescence under GFP excitation light (**Figure 2A**). PCR results confirmed that *Psa*-GFP harbors the pDSK-GFPuv plasmid (**Figure 2B**). To improve the operability of *Psa* inoculation and increase the repeatability of inoculating explants, we developed an efficient inoculation method for kiwifruit branches. This involved making a wound in the middle of an 8 cm kiwifruit branches using a scalpel, followed by inoculating 1 μL of bacterial suspension (OD_600_ = 0.8) at the wound site (**Figure 2C**). The inoculation sites and the base of the branches were wrapped with sterile cotton soaked in water, and the branches was placed in a humidity condition at 25 °C for *Psa*-GFP infection (**Figure 2D**). 14 days after inoculation, the cotton was removed, along with 0.5 cm^2^ of the epidermis around the inoculation site. Under daylight, the inoculation site of rootstock ‘DE’ appeared healthy and green. Under GFP excitation light, only red autofluorescence from chlorophyll was observed, with no visible GFP fluorescence (**Figure 2E**). In contrast, the inoculation site on the commonly used scion, *A. chinensis* ‘HY’, exhibited a dark brown color. Additionally, under GFP excitation light, strong green fluorescence from *Psa*-GFP was observed (**Figure 2E**). Furthermore, both the longitudinal and cross-sections of *A. chinensis* ‘HY’ branches inoculated with *Psa*-GFP displayed green fluorescence under handheld excitation light and fluorescence microscopy. In contrast, no noticeable green fluorescence was observed in the inoculated *A. valvata* or in the uninfected controls, indicating that *A. valvata* strongly restricts the colonization and proliferation of the pathogen, thereby demonstrating its high resistance to *Psa* (**Figure 2F**). The cutting experiment showed that the survival rate of *A. valvata* cuttings in vermiculite exceeded 80%, while those of *A. chinensis* ‘HY’, *A. chinensis* ‘DH’, and *A. chinensis* ‘XX’ were below 1% (**Figures 2G, I**; **Supplementary Figure 1**). The hydroponic results were similar to the cutting results, with *A. valvata* cuttings surviving in water at a rate of over 90%, while all cuttings of *A. chinensis* ‘HY’, *A. chinensis* ‘DH’, and *A. chinensis* ‘XX’ ultimately died, indicating *A. valvata* is resistant to flood (**Figures 2H, J**).

### 3.2 Establishment of the root genetic transformation system for *A. valvata*

**Figure 3:**
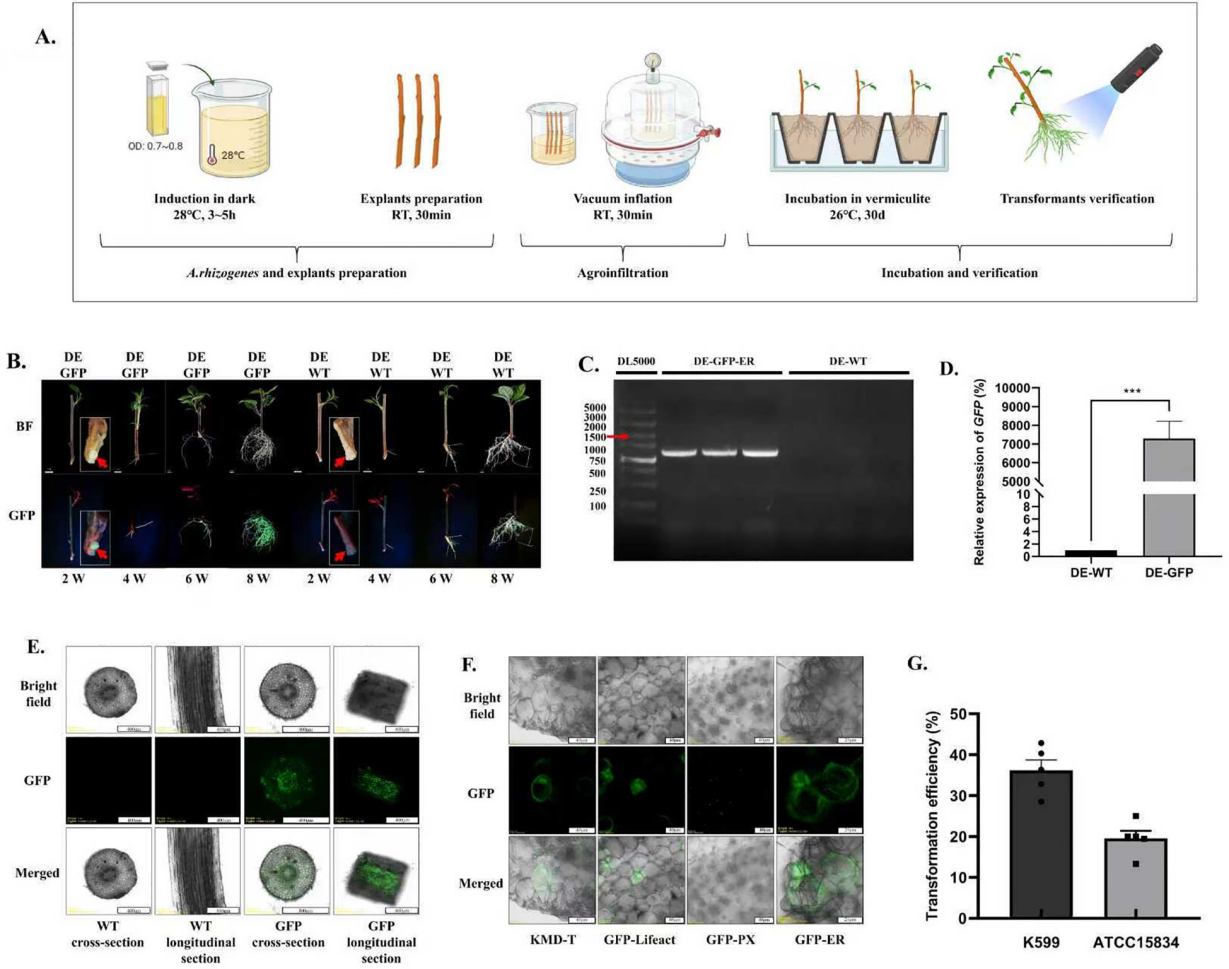
A*. rhizogenes*-mediated transformation system in *A. valvata*. **(A)** Schematic diagram of *A. rhizogenes*-mediated transformation system in *A. valvata*. **(B)** Transgenic callus or roots verification. The position indicated by the arrow is the transgenic callus. **(C)** PCR verification of transgenic roots. ‘DE-GFP-ER’ represent transgenic root, ‘DE-WT’ represent wild-type. **(D)** RT-qPCR verification of transgenic root and wild type. **(E)** Validation of transgenic hairy roots using confocal microscopy. **(F)** Observation of GFP localization in different organelles. ‘KMD-T’,’GFP-Lifeact’, ‘GFP-PX’, and ‘GFP-ER’ represent plasmids containing GFP targeted tomicrotubules, microfilaments, peroxisomes, and the endoplasmic reticulum, respectively. **(G)** Genetic transformation efficiency analysis of kiwifruit branches mediated by *A. rhizogenes* strains K599 and ATCC15834 bypassing tissue culture.

**Figure 4:**
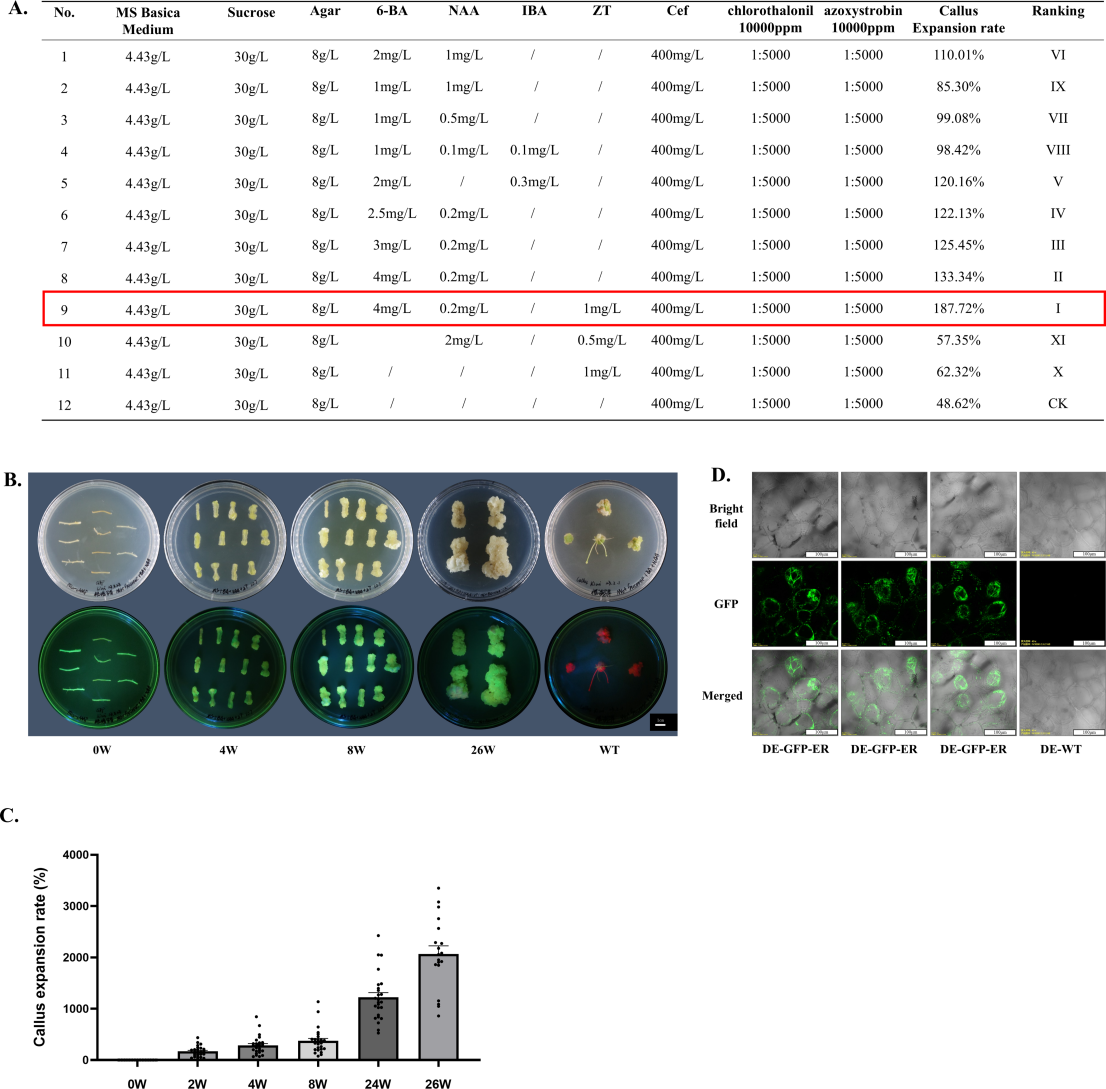
Callus induction of transgenic hairy roots of *A. valvata*. **(A)** Callus induction efficiency analysis of transgenic hairy roots cultured on media with different hormone ratios. **(B)** Morphological detection of callus derived from transgenic hairy roots at different time points. **(C)** Callus growth rate analysis of transgenic hairy roots cultured on medium No. 9. **(D)** Observation of callus tissue using confocal microscopy.

Based on the high survival rate of *A. valvata* cuttings, *A. rhizogenes*-mediated genetic transformation system for hairy roots bypassing tissue culture was developed. The main procedures include the preparation of *A. rhizogenes* and explants, agroinfiltration, incubation and screening (**Figure 3A**). 2 weeks after transformation, transgenic callus formed on branches, and by 4 weeks, transgenic hairy roots developed. By 8 weeks, the transgenic hairy roots were well-developed, with a transformation success rate of 42% (**Figure 3B**). At the corresponding time points, untreated control branches also developed normal callus and hairy roots, but without GFP fluorescence (**Figure 3B**). PCR results showed that the fluorescent hairy roots contained the characteristic fragment of the GFP-ER plasmid in their genome, further confirming the success of the genetic transformation (**Figure 3C**). RT-qPCR results revealed that the *GFP* was highly expressed in transgenic roots (**Figure 3D**). Confocal laser scanning microscopy results showed that all cells of the transgenic hairy roots emitted green fluorescence under excitation light, while no fluorescence was observed in any cells of the wild-type hairy roots, indicating a low probability of non-transgenic cell chimerism in the transgenic hairy roots (**Figure 3E**). Using this transformation system, we successfully achieved the genetic transformation of plasmids containing GFP localized to different organelles in *A. valvata*. Confocal microscopy revealed that the corresponding organelles exhibited stable and intense green fluorescence, demonstrating the method’s suitability for the genetic transformation of various types plasmids (**Figure 3F**). Additionally, a comparative analysis of the genetic transformation efficiency of two *A. rhizogenes* strains, K599 and ATCC15834, was conducted. The results demonstrated that the transformation efficiency of K599 (36%) was significantly higher than that of ATCC15834 (20%) (**Figure 3G**). Therefore, K599 was used for subsequent hairy root transformation experiments. Cultivation of transgenic hairy roots on MS medium with varying hormone concentrations revealed that the medium containing 4 mg/L 6-BA, 0.2 mg/L NAA, and 1 mg/L ZT was most effective for callus formation, though none of the tested media induced shoot formation (with cefotaxime, chlorothalonil, and azoxystrobin used to inhibit bacterial and fungal growth) (**Figure 4A**). Under this induction condition, callus formed on the entire transgenic hairy root explant within 4 weeks and continued to increase in size over time, with GFP fluorescence consistently present (**Figures 4B, C**). Confocal microscopy revealed that all cells in the detected callus tissues displayed stable and intense green fluorescence, further indicating a low likelihood of non-transgenic cell chimerism in the transgenic tissues (**Figure 4D**).

### 3.3 Application of *A. rhizogenes*-mediated transformation system in *A. chinensis* ‘HY’

**Figure 5:**
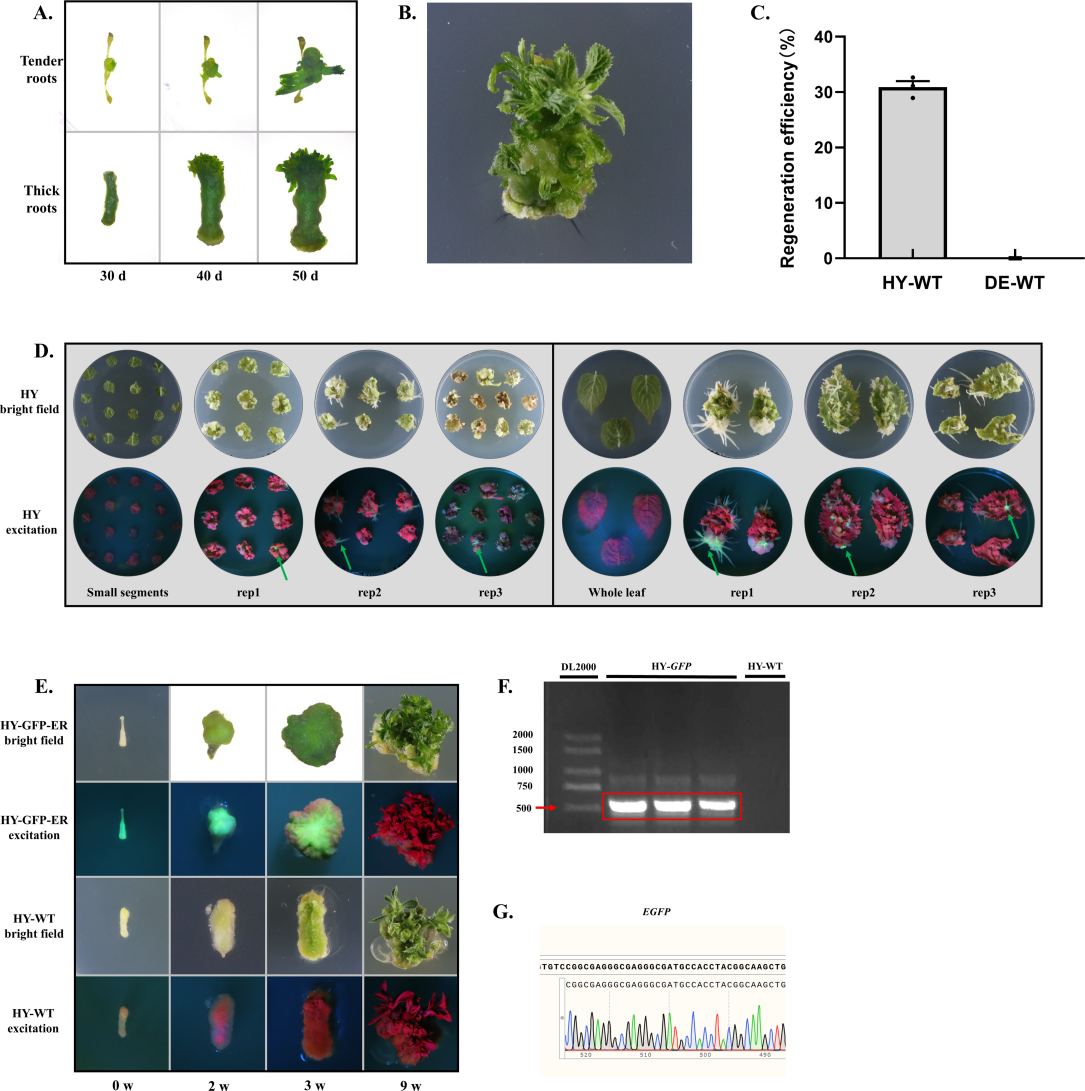
A *rhizogenes*-mediated transformation system in *A. chinensis* ‘HY’. **(A)** Shoot induction of wild-type hairy roots. Tender roots indicate newly formed hairy roots, while thick roots represent more mature root segments. **(B)** Leaf growth status observed two months after shoot induction culture of wild-type roots. **(C)** Shoot induction efficiency analysis from wild-type roots of *A. chinensis* ‘HY’ and *A. valvata*. **(D)** *A. rhizogenes*-mediated hairy roots genetic transformation from *A. chinensis* ‘HY’ leaves through tissue culture. **(E)** Shoot induction in transgenic hairy roots. ‘HY-GFP-ER’ represents transgenic roots, while ‘HY-WT’ represents wild-type roots. **(F)** PCR verification of transgenic roots. **(G)** DNA sequencing verification of transgenic roots.

Given the significant differences in root-induced shoot regeneration efficiency among species, this study also conducted root induction and shoot regeneration analysis in another kiwifruit species, *A. chinensis* ‘HY’. The results showed that when the most efficient callus inducing medium in *A. valvata* was used (**Figure 4A**, medium 9), both tender roots and thick roots of *A. chinensis* ‘HY’ could form shoots within approximately 30 days (**Figure 5A**). It is worth noting that tender roots are prone to necrosis at both ends during tissue culture, resulting in transgenic shoots mainly forming in the central region, with fewer shoots overall (**Figure 5A**). In contrast, thick roots showed little necrosis during the tissue culture process, and callus formed along the entire root segment, leading to a higher number of shoots (**Figure 5A**). After approximately two months of root culture, leaves developed normally and were suitable for various physiological measurements (**Figure 5B**). Comprehensive analysis of three independent shoot regeneration experiments revealed that *A. chinensis* ‘HY’ had a successful shoot induction rate of 31%, whereas *A. valvata* showed no successful regeneration, indicating that the root-induced shoot regeneration efficiency of *A. chinensis* ‘HY’ is significantly higher than that of *A. valvata* (**Figure 5C**). Since *A. chinensis* ‘HY’ branches are difficult to survive under cutting conditions, leaf explants were used for *A. rhizogenes*-mediated transformation in this study. Leaf explants of *A. chinensis* ‘HY’ were either whole leaves or leaves cut into approximately 0.5 mm diameter segments (**Figure 5D**). After infiltration with *Agrobacterium*, the explants were placed on co-culture medium (2 days, 24 °C, dark) and then transferred to root induction medium (24 °C, dark) to obtain transgenic hairy roots (**Supplementary Figure 2**). After approximately two months of culture, whole leaf explants showed a significantly higher rate of hairy root formation compared to the 0.5 mm diameter leaf segments (**Figure 5D**). Using the shoot induction medium (**Figure 4A**, medium 9), transgenic root segments were induced, showing significant callus growth and normal chlorophyll synthesis after 2–3 weeks (**Figure 5E**). After 9 weeks, numerous leaves formed, with a formation rate comparable to that of the wild type (**Figure 5E**). It is important to note that under handheld excitation light, the GFP fluorescence in the callus formed by transgenic roots gradually weakened as chlorophyll increased, and only red chlorophyll autofluorescence was observed in the induced leaves (**Figure 5E**). PCR and DNA sequencing results confirmed that the transgenic leaves contained the *GFP* gene, while the wild-type leaves did not (**Figures 5F, G**).

### 3.4 The developed genetic transformation system in *A. valvata* is applicable to *M. rubra*

**Figure 6:**
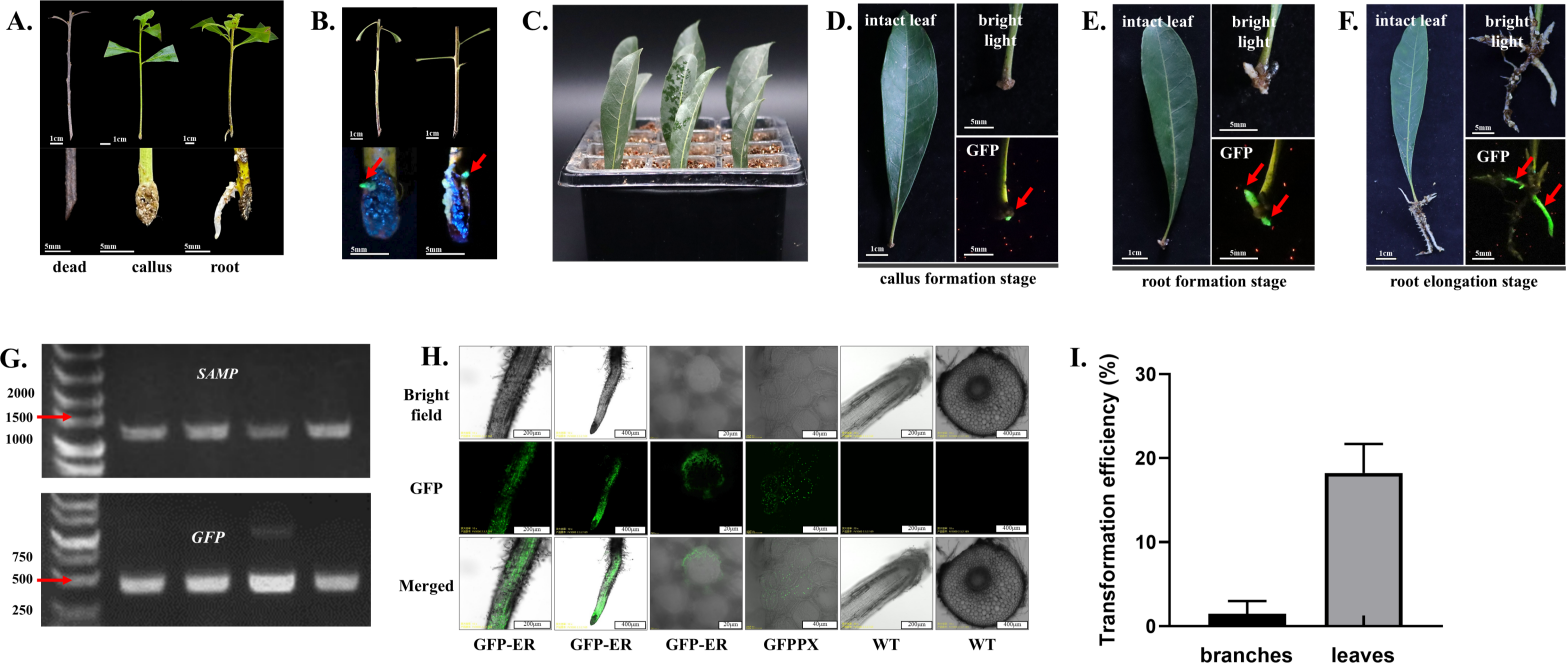
A *rhizogenes*-mediated transformation system in *M. rubra*. **(A)** Rooting ability analysis in *M. rubra* cuttings. **(B)** A. rhizogenes-mediated genetic transformation of *M. rubra* branches. Fluorescent spots indicate transgenic callus. **(C)** Schematic diagram of *M. rubra* leaf cutting condition after Agro-infiltration. **(D)** Observation of transgenic callus formation at the petiole of *M. rubra* (3–4 Weeks). **(E)** Observation of transgenic hairy root formation at the petiole of *M. rubra* (4–6 Weeks). **(F)** Observation of the elongation stage of transgenic hairy roots at the petiole of *M. rubra*. **(G)** PCR verification of transgenic roots. ‘SAMP’ and ‘GFP’ are DNA sequence containing in GFP-ER plasmid. **(H)** Confocal microscopy observation of transgenic roots and wild-type roots. ‘GFP-ER’ and ‘GFP-PX’ represent transgenic roots containing ‘GFP-ER’ and ‘GFP-PX’ plasmid. ‘ WT ‘ represents the wild-type roots. **(I)** Efficiency analysis of *A. rhizogenes*-mediated genetic transformation in branches or leaves of *M. rubra*.

Without the addition of exogenous rooting hormones, the rooting rates of *M. rubra* branch cuttings was only 2% (**Figure 6A**). Using the transformation system developed in this study, *M. rubra* branch cuttings were successfully transformed to produce GFP-fluorescent transgenic callus (**Figure 6B**). However, the callus failed to develop into complete root systems, and all transformed branches eventually died, consistent with the low survival rate observed in the cutting experiment. Since *M. rubra* leaf cuttings displayed a higher survival rate, subsequent genetic transformation experiments were conducted on leaves. Agroinfiltrated leaves were cultured in vermiculite at 24-26 °C under a 14h/10h (light/dark) photoperiod (**Figure 6C**). 3–4 weeks after agroinfiltration, GFP-fluorescent callus began to form at the base of the petioles (**Figure 6D**). After 4–6 weeks, the transgenic callus started to differentiate into hairy roots (**Figure 6E**). Over time, the transgenic hairy roots continued to grow, and the leaves remained healthy (**Figure 6F**). 10 weeks after agroinfiltration, the transgenic roots grew over 5 cm (**Supplementary Figure 3**). Although non-transgenic roots may also form, the GFP allowed for quick distinction between transgenic and non-transgenic roots using a handheld excitation light source. PCR results revealed that the sequences in the plasmid of GFP-ER were existed in the genome of transgenic hairy roots (**Figure 6G**). Confocal microscopy confirmed that all the transgenic hairy root cells emitted GFP fluorescence, indicating a low probability of chimerism with non-transgenic cells (**Figure 6H**). The transformation success rate of branch cuttings was less than 2%, while leaf transformation achieved an 18.23% success rate (**Figure 6I**). Given the easier availability and high transformation efficiency, leaves are the preferred target for genetic transformation in *M. rubra*.

### 3.5 Analysis of HMs toxicity on the root system of *A. valvata*

**Figure 7:**
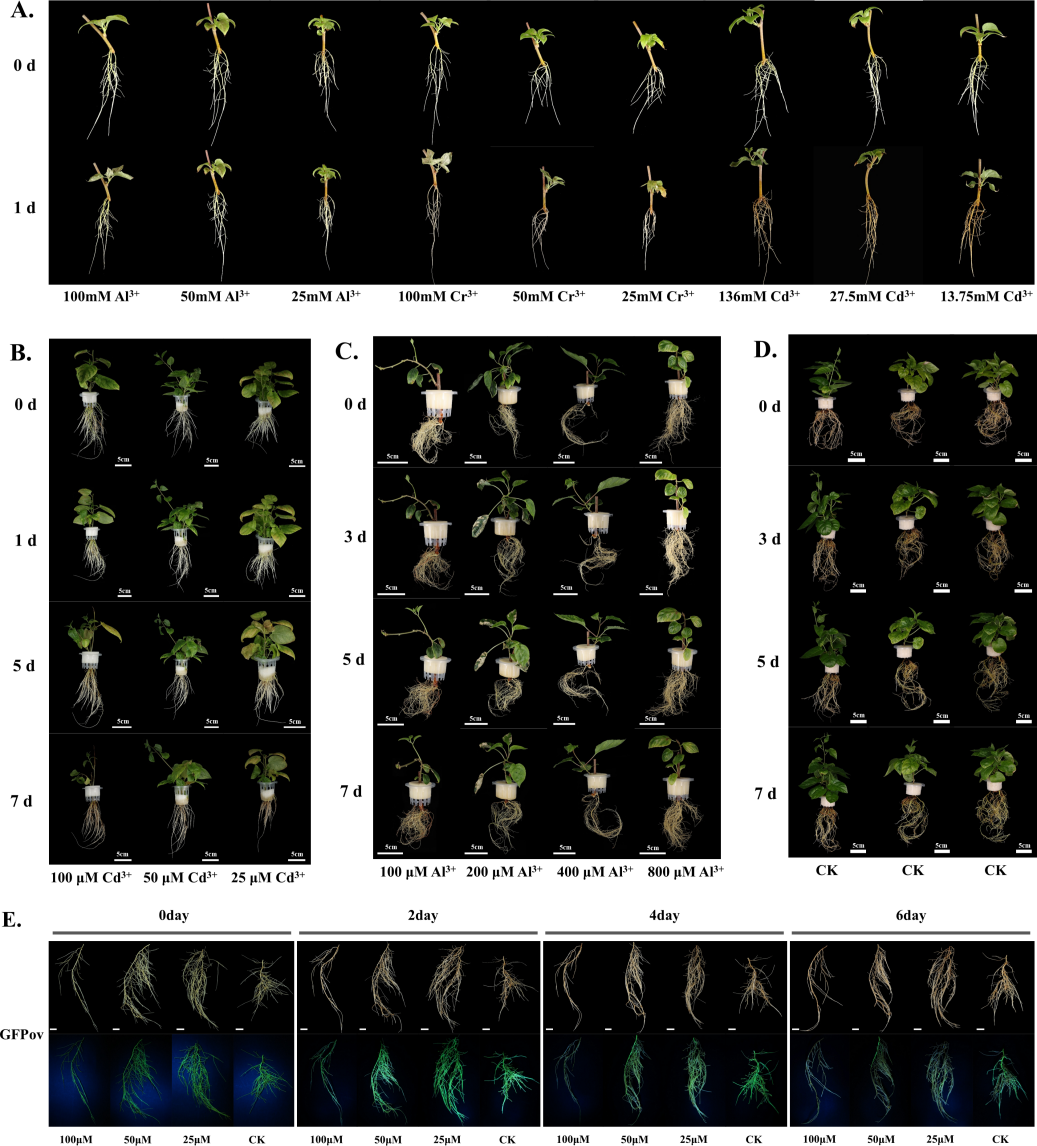
Phenotypic and physiological evaluation of heavy metal toxicity on the root system of *A. valvata*. **(A)**. Toxicity analysis of high concentrations of Al³^+^, Cr³^+^, and Cd²^+^ on *A. valvata* under short-term exposure. **(B-D)**. Toxicity analysis of Cd²^+^ (25-100 µM) and Al³^+^ (100-800 µM) on *A. valvata* at 0, 1, 5, and 7 days, with a hydroponic solution free of heavy metals serving as the control group (CK). **(E)**. Effects of varying concentrations of Cd²^+^ on transgenic root systems *in vitro*.

To assess *A. valvata*’s sensitivity to heavy metals, the plants with high concentrations of Al^3+^, Cr^3+^, and Cd^2+^ were treated. Sensitivity was ranked as Cd^2+^ > Cr^3+^ > A^l3+^. After one day of Cd^2+^ treatment at concentrations above 10 mM, the roots turned brown, and the leaves began to necrose (**Figure 7A**). Cr^3+^ treatment caused leaf necrosis without affecting root color, while Al^3+^ treatment had no significant impact on either roots or leaves (**Figure 7A**). When the Cd^2+^ concentration was reduced to 25-100 μM and treated for 1 day, no significant effects were observed on the roots or leaves (**Figure 7B**). After 7 days, root color darkened and leaf necrosis developed, indicating a severe impact of Cd^2+^ on cells at this concentration (**Figure 7B**). Treatment with higher concentrations of Al^3+^ (100-800 μM) for 7 days did not significantly affect the phenotype of kiwifruit roots and leaves (**Figure 7C**). Even when the Al^3+^ concentration was increased to 1600 μM and the treatment duration extended to 15 days, the phenotype of roots directly exposed to Al^3+^ remained unaffected (**Supplementary Figure 4**). The control group exhibited normal growth and development of roots and leaves under hydroponic conditions, indicating that *A. valvata* is sensitive to Cd^2+^ but tolerant to Al^3+^. When noticeable changes in the roots and leaves phenotypes occur, it typically indicates severe cellular damage. Therefore, the initial cellular physiological impact occurs much earlier than the appearance of external phenotypic changes. To analyze the physiological impact of Cd^2+^ on kiwifruit cells, we constructed a large number of GFP-overexpressing hairy roots using the developed genetic transformation system. These roots were treated with different concentrations of Cd^2+^ *in vitro*, and after 2 days of 100 μM Cd^2+^ treatment, most roots showed a drastic decrease in fluorescence intensity, which nearly vanished after 4 days (**Figure 7E**). Treatment with 25 μM and 50 μM Cd^2+^ for 4 days also caused marked fluorescence reduction, which almost completely disappeared after 6 days (**Figure 7E**).

### 3.6 Cd^2+^ stress influence the hormone homeostasis and gene transcription in *A. valvata*

**Figure 8:**
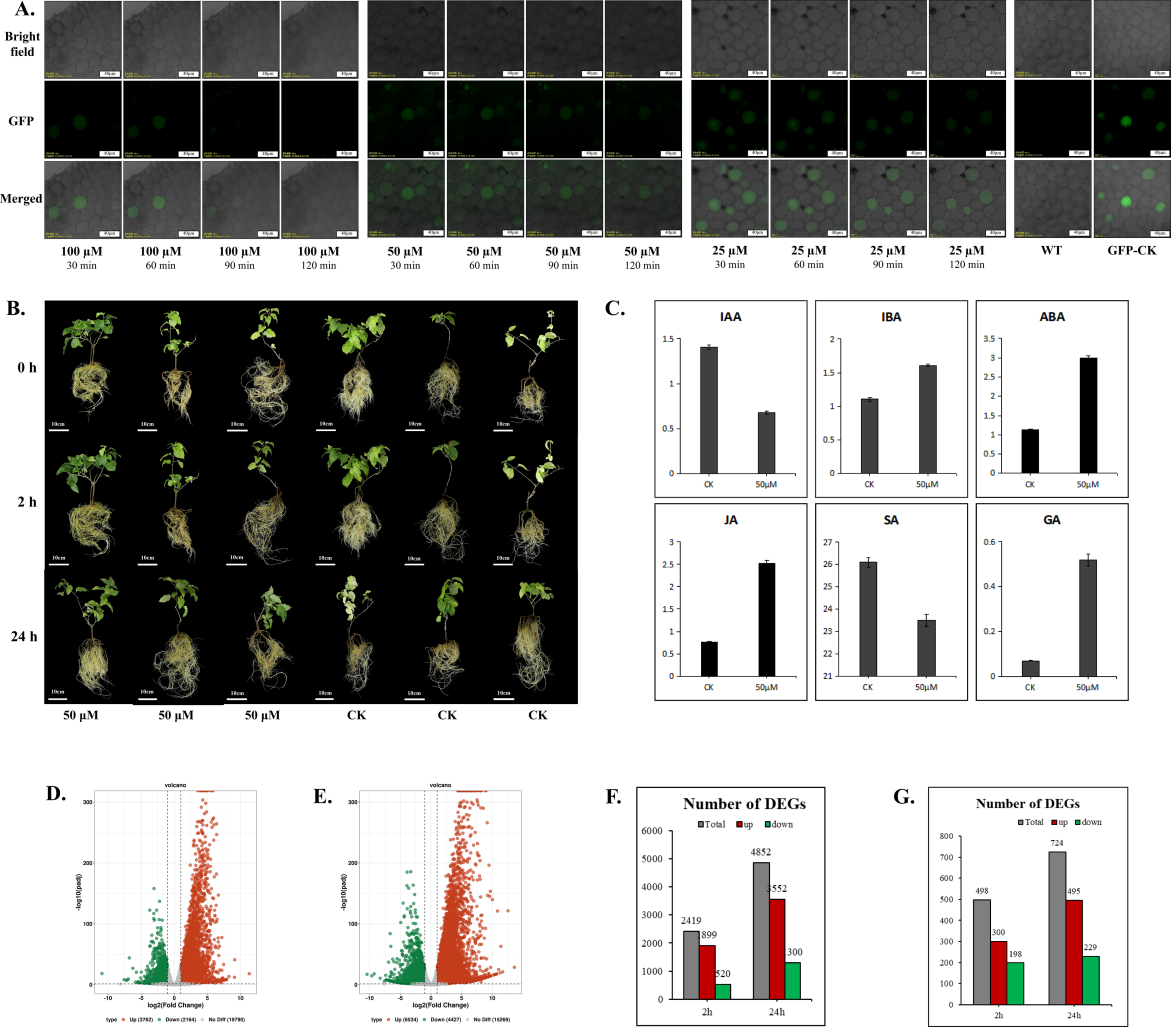
Transcriptomic and hormonal responses of *A. valvata* roots to Cd²^+^ stress. **(A)** Confocal microscopy analysis of GFP fluorescence intensity changes over time in *GFP*-overexpressing transgenic root cells under different concentrations of Cd²^+^ treatment. **(B)** Plants used for RNA-seq and hormone content detection. ‘0h’, ‘2h’, and ‘24h’ represent plants treated with 50 µM Cd²^+^ for 0h, 2h, and 24h, respectively. **(C)** Hormone content detection in roots after 24h of treatment with 50 µM Cd²^+^, with untreated roots serving as the control group (CK). DEGs in roots after 2h **(D)** and 24h **(E)** of treatment with 50 µM Cd²^+^. **(F)** Number of DEGs with a fold change exceeding 4. ‘2h’ and ‘24h’ represent the number of DEGs with a fold change greater than 4 in roots treated with 50 µM Cd²^+^ for 2h and 24h, respectively, compared to the untreated group. **(G)** The number of transcription factors showing significant differential expression after 2h and 24h of treatment with 50 µM Cd²^+^.

**Figure 9:**
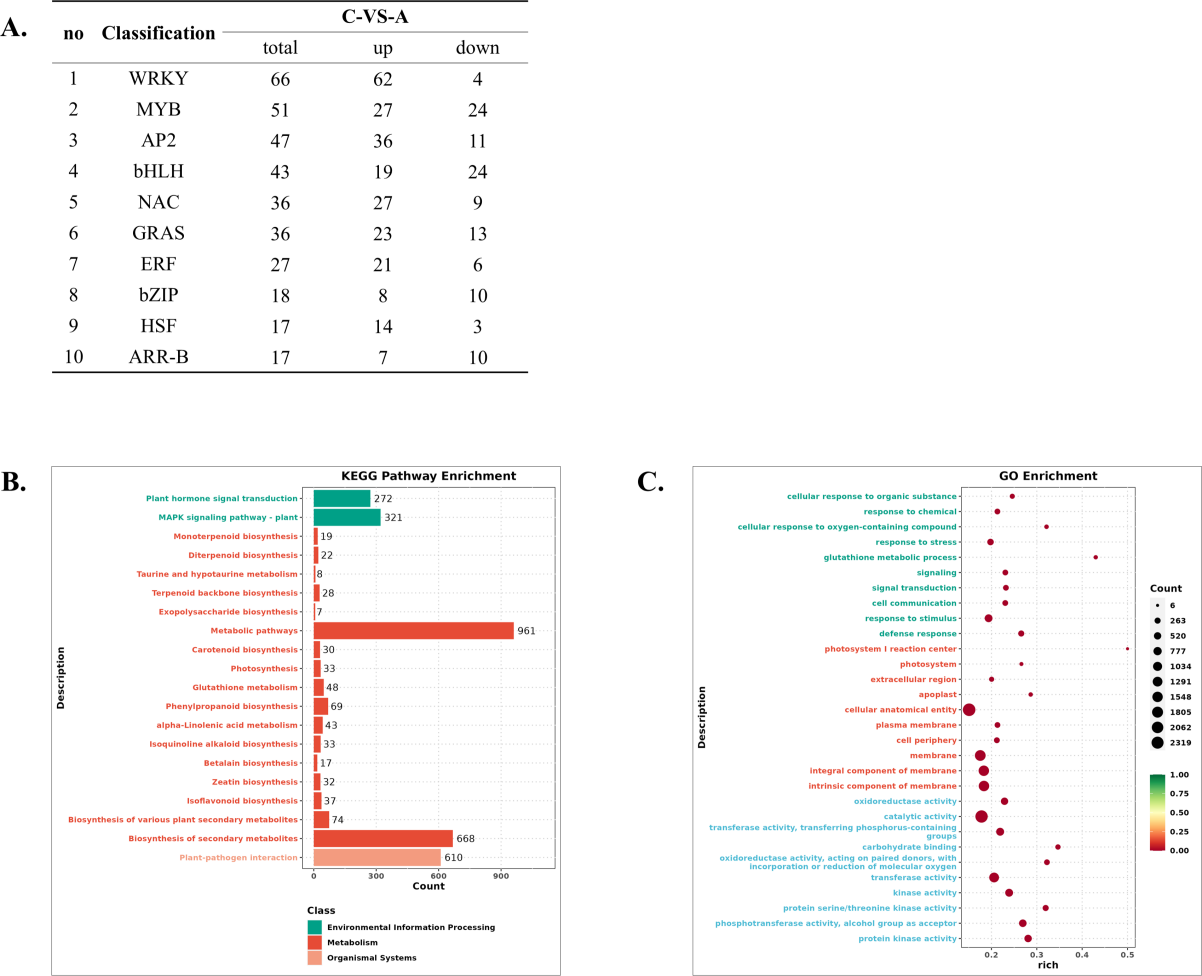
Cd²^+^ stress influence the hormone homeostasis and gene transcription in *A. valvata*. **(A)** Top ten transcription factor families with the highest number of DEGs in *A. valvata* roots treated with 50 µM Cd²^+^ for 2h. **(B)** KEGG pathway enrichment analysis of DEGs in roots treated with 50 µM Cd²^+^ for 2h. **(C)** GO enrichment analysis of DEGs in roots treated with 50 µM Cd²^+^ for 2h.

Confocal microscopy showed that treatment with 100 μM Cd^2+^ for 90 minutes visibly reduced GFP fluorescence in transgenic hairy roots, while 25 μM and 50 μM Cd^2+^ treatments for 120 minutes also caused marked fluorescence reduction, indicating that Cd^2+^ at these concentrations can impact kiwifruit cellular functions within 120 minutes (**Figure 8A**). To assess the effect of Cd^2+^ on hormone levels and gene transcription in kiwifruit roots, we treated plants with 50 μM Cd^2+^ and collected root samples at 0h, 2h, and 24h (**Figure 8B**). Because the biosynthesis and robust physiological accumulation of hormone metabolites typically lag behind rapid initial transcriptional signaling, we selected the 24h time point to capture the peak hormone-mediated defense responses. Hormone analysis showed that 24 h treatment with 50 μM Cd^2+^ significantly increased IBA, ABA, JA, and GA contents, while IAA and SA contents decreased, indicating Cd^2+^ stress disrupted hormone homeostasis in kiwifruit roots (**Figure 8C**). However, despite these significant hormonal shifts within 24 h, macroscopic changes in root architecture—such as alterations in root length and the number of lateral roots—were not yet visually evident at this early stage (**Figure 8B**). These morphological adaptations typically require a longer period of continuous Cd²^+^ exposure to manifest. Transcriptome analysis showed that after 2 h of Cd^2+^ treatment, 5,926 DEGs were identified, including 3,762 upregulated and 2,164 downregulated genes (**Figure 8D**, **Supplementary Table 3**). Of these, 2,419 genes had a more than fourfold change (|log2FoldChange| > 2), with 1,899 upregulated and 520 downregulated (**Figure 8F**, **Supplementary Table 3**). After 24 hours, 10,961 DEGs were identified, including 6,534 upregulated and 4,427 downregulated genes (**Figure 8E**, **Supplementary Table 4**). Among these, 4,852 genes exhibited more than a fourfold change, with 3,552 upregulated and 1,300 downregulated (**Figure 8F**, **Supplementary Table 4**). Transcription factors act as master switches in plant stress responses, and analyzing their dynamics reveals the core regulatory networks governing downstream defense mechanisms. After 2 h of Cd^2+^ treatment, 300 transcription factors were upregulated and 198 downregulated. After 24 h, 495 were upregulated and 229 downregulated (**Figure 8G**; **Supplementary Tables 5**, **S6**). The number of upregulated transcription factors was significantly higher than downregulated, aligning with the overall trend in the transcriptome, where upregulated genes outnumbered downregulated ones. After 2 hours of Cd^2+^ treatment, 7 of the top 10 transcription factor families by DEG count contained more upregulated than downregulated genes. Families with over 30 DEGs included WRKY, MYB, AP2, bHLH, NAC, and GRAS (**Figure 9A**). KEGG pathway enrichment analysis revealed that the five most significantly affected pathways were “Metabolic pathways”, “Biosynthesis of secondary metabolites”, “Plant-pathogen interaction”, “MAPK signaling pathway”, and “Plant hormone signal transduction” (**Figure 9B**; **Supplementary Tables 7**, **S8**). GO enrichment analysis revealed that the most significantly affected categories were “cellular anatomical entity”, “catalytic activity”, “membrane”, “intrinsic component of membrane”, and “integral component of membrane” (**Figure 9C**; **Supplementary Tables 9**, **S10**). Additionally, many DEGs were significantly enriched in “response to chemical”, “response to stress”, “response to stimulus”, “transferase activity”, and “kinase activity” (**Figure 9C**). The generation of reactive oxygen species (ROS) and inhibition of enzymatic activity represent two critical aspects of Cd-induced physiological alterations in plant cells. Heat shock proteins (HSPs), glutathione (GSH), catalase (CAT), and superoxide dismutase (SOD) play pivotal roles in ROS detoxification or maintenance of protein functionality. Transcriptomic analysis revealed significant transcriptional changes in response to Cd^2+^ treatment: after 2h exposure, 17 HSPs showed upregulated expression (**Supplementary Table 11**), which increased to 30 HSPs (29 upregulated) following 24h treatment (**Supplementary Table 12**). Similarly, GSH system-related genes exhibited substantial changes, with 26 glutathione-S-transferase (GST) genes (24 upregulated) at 2h (**Supplementary Table 13**) and 50 GST genes (42 upregulated) at 24h (**Supplementary Table 14**). Glutaredoxin (GRX) genes showed 9 upregulated transcripts at 2h (**Supplementary Table 15**) and 17 transcripts (14 upregulated) at 24h (**Supplementary Table 16**). Both adenosine 5’-phosphosulfate reductase (APR), which is involved in glutathione synthesis, and CAT showed significant upregulation at both time points, whereas SOD genes displayed an equal number of up- and down-regulated genes (**Supplementary Tables 17**, **S18**). Furthermore, analysis of three ion transport-related gene families (ABC transporter family, Nramp gene family, and MATE efflux family protein) revealed substantial transcriptional regulation with numerous up- and down-regulated genes following both 2h and 24h Cd^2+^ treatments (**Supplementary Tables 17**, **S18**).

## 4 Discussion

Kiwifruit is an important fruit crop that is frequently affected by both biotic and abiotic stresses during its growth, including canker, HMs, and waterlogging, which severely threaten its productivity and quality. Rootstock selection is a key factor in orchard management, as it influences the environmental tolerance, nutrient accumulation, growth, and fruit quality of scion varieties (Dubey and Sharma, 2016; Wang et al., 2017; Faria-Silva et al., 2020). However, the lack of a genetic transformation system in kiwifruit rootstocks has hindered in-depth research on their mechanisms for responding to these stresses. Since rootstocks function primarily through their roots, establishing an efficient genetic transformation system for roots is crucial for rootstock research. This study focuses on the commonly used kiwifruit rootstock, *A. valvata*, demonstrating its advantages such as canker resistance, waterlogging tolerance, high survival rate in cuttings, and strong tolerance to Al^3+^. These characteristics make it an ideal rootstock variety for establishing the root genetic transformation system and conducting related phenotypic studies. Therefore, this study established an *A. rhizogenes*-mediated hairy root genetic transformation system for *A. valvata*, also applicable to *M. rubra*. Using this system, the effects of Cd²^+^ on kiwifruit roots were explored.

### 4.1 *A. valvata* is resistant to canker, Al^3+^, and waterlogging

Bacterial canker, caused by *Psa*, severely damages kiwifruit and can lead to tree death. It is regarded as one of the primary limiting factor in kiwifruit production (Vanneste, 2017; Sawada and Fujikawa, 2019). *A. chinensis* and *A. deliciosa* are the primary kiwifruit species affected by *Psa*, with infections also reported in the wild species *A. arguta* and *A. kolomikta* (Ushiyama et al., 1992; Marcelletti et al., 2011; Kim et al., 2016). Therefore, identifying kiwifruit species or genotypes resistant to canker is crucial for developing effective control strategies against the disease. This study demonstrates that *A. valvata* exhibits high resistance to bacterial canker by inhibiting the proliferation and spread of *Psa* in branches. In addition to its high resistance to canker, *A. valvata* also exhibits strong tolerance to Al³^+^. Al toxicity, a major constraint on agricultural productivity, occurs in acidic soils (pH < 5) where Al is solubilized as Al³^+^ (Driscoll, 1985; Von Uexküll and Mutert, 1995). This ion disrupts signaling, induces ROS overproduction, damages membranes, and inhibits root growth (Yamamoto et al., 2002; Rengel and Zhang, 2003; Ma, 2007; Sun et al., 2014; Kochian et al., 2015). With 30% of global land and nearly half of arable land potentially impacted by acidic soils, Al toxicity poses a significant challenge to global agricultural productivity (Wang et al., 2015; Shetty et al., 2021). For example, 100 µM Al^3+^ can severely impair the physiological functions of *Carya cathayensis* (Zeng et al., 2023). In contrast, at the same Al³^+^ concentration, *A. valvata* exhibits no significant physiological changes. Even at eight times the concentration (800 µM), *A. valvata* continues to grow normally, demonstrating its high tolerance to Al³^+^. Species similar to *A. valvata*, such as *Camellia* spp., *Vochysia tucanorum*, *Quercus serrata*, *Symplocos paniculata*, *Coffea arabica*, and *Melastoma malabathricum*, are Al-hyperaccumulators and are capable of thriving in acidic soils (Hajiboland et al., 2013; Bojórquez-Quintal et al., 2014; Schmitt et al., 2016; Liu et al., 2020; Sun et al., 2020; Bressan et al., 2021). Over the decades, numerous studies have highlighted the beneficial effects of aluminum (Al) on plants, yet its biological significance at the cellular level remains unproven (Arasimowicz-Jelonek et al., 2014; Muhammad et al., 2019; Liu et al., 2020; Sun et al., 2020). Establishing an efficient genetic transformation system is crucial for investigating the mechanisms of plant responses to specific heavy metal stress at the cellular and molecular levels. Waterlogging and similar adverse conditions severely hinder horticultural crop growth, causing substantial losses. Each year, over 1,700 Mha of land is affected by waterlogging, leading to root hypoxia, metabolic disruption, and often plant death (Voesenek and Sasidharan, 2013). Recent studies have revealed that *A. valvata* exhibits significantly higher tolerance to waterlogging stress compared to *A. chinensis* (Bai et al., 2019; Li et al., 2022). In this study, *A. valvata* grow healthily under hydroponic conditions, whereas *A. chinensis* cannot develop a root system, further confirming the higher waterlogging stress tolerance of *A. valvata*. Given *A. valvata*’s strong resistance to the aforementioned biotic and abiotic stresses, establishing an efficient genetic transformation system is crucial to enable more comprehensive research.

### 4.2 The *A. rhizogenes*-mediated transformation system is applicable to *A. valvata*

*A. valvata* is commonly used as rootstock in kiwifruit production, where it primarily functions through its roots. The *A. tumefaciens*-mediated genetic transformation system relies on tissue culture, typically involving the generation of transgenic shoots first, followed by the induction of transgenic roots from these shoots (Kumar et al., 2010; Zhang et al., 2022). This method is high cost and time-consuming to obtain transgenic roots. Unlike *A. tumefaciens*, *A. rhizogenes*-mediated genetic transformation does not rely on tissue culture and can produce transgenic roots in a much shorter time frame (Meng et al., 2019; Cao et al., 2023). The recalcitrance of many woody species to genetic transformation typically stems from their poor cellular dedifferentiation capacity. Our findings suggest that the exceptionally high intrinsic rooting ability of *A. valvata* cuttings provides a highly competent cellular environment at the wound site, facilitating rapid *rol* gene integration and expression. Specifically, there is a strong biological correlation between natural cutting rooting capacity and *A. rhizogenes* induction efficiency. Both processes rely on the presence of highly competent cells at the wound site capable of rapid dedifferentiation; thus, genotypes that readily root from cuttings are inherently more receptive to the *rol* genes transferred by *A. rhizogenes*. By bypassing the lengthy, genotype-dependent tissue culture regeneration processes, this in planta composite system shifts the research paradigm in woody horticultural crops from laborious methodological optimization to immediate, high-throughput functional validation of stress-responsive genes directly within the functional rootstock. This study also performed genetic transformation using plasmids containing GFP targeted to different organelles, followed by observation of the corresponding organelle morphologies. Moreover, this method is also effective for the genetic transformation of the more challenging *M. rubra*. Notably, the explant materials for this transformation system, whether branches or leaves, can be conveniently collected directly from the field without the need for sterilization or disinfection. This technological system enables efficient functional studies of target genes in the roots, facilitating in-depth research on the regulatory mechanisms of rootstock disease resistance and stress tolerance. Confocal microscopy revealed that almost all cells in the transgenic roots emitted GFP-derived green fluorescence, and the transgenic callus tissues induced from these roots also displayed green fluorescence in all cells. This indicates a low likelihood of chimeric tissues with wild-type cells in the transgenic roots, ensuring high precision for target gene functional studies in roots. In addition, this study successfully applied the technology to *A. chinensis* ‘HY’ and achieved shoot regeneration from transgenic hairy roots. In 2024, researchers developed a method to induce shoot formation from transgenic roots of *A. chinensis* ‘HY’, which holds significant importance for studying gene function in this species. However, their approach required two sequential steps: first, inducing callus formation using a callus induction medium, followed by shoot induction using a shoot induction medium. In contrast, the method established in this study enables shoot formation in a single step within 4–5 weeks, offering advantages such as simplified operation, short experimental cycles, and reduced costs.

### 4.3 Genetic transformation facilitates in-depth research on the effects of Cd²^+^ on *A. valvata*

Cd toxicity in plants is often initially assessed through visible physiological changes, but this approach typically takes days or even weeks to reveal its toxic effects on plant tissues. In this study, *A. valvata* displayed significant physiological phenotypic changes only after over a week of treatment with 100 µM Cd²^+^. To bypass this time lag, tracking GFP fluorescence intensity serves as a rapid and highly sensitive proxy for assessing cell viability (Steff et al., 2001; Strebel et al., 2001). Therefore, GFP-overexpressing transgenic roots of *A. valvata* were generated and confocal microscopy was employed to track GFP fluorescence intensity changes in root cells over time after exposure to different concentrations of Cd²^+^. The microscopy results revealed that a 2-hour treatment with 100 µM Cd²^+^ significantly reduced GFP fluorescence intensity in transgenic root cells, occurring well before obvious physiological changes were observed on the plant’s surface. Currently, researchers have conducted extensive transcriptomic sequencing of plant species treated with Cd (Zhou et al., 2016; Feng et al., 2018; Huang et al., 2019; Xu et al., 2019). In this study, transcriptomic sequencing was performed on the roots of *A. valvata* at 0h, 2h, and 24h following Cd treatment based on GFP fluorescence intensity changes. Rather than merely restating the extensive transcriptomic alterations, our rapid GFP-quenching assay, coupled with transcriptomic profiling, reveals that *A. valvata* root cells undergo massive and acute cellular reprogramming within merely two hours of Cd²^+^ exposure. The rapid functional enrichment in MAPK cascades and profound shifts in hormone signal transduction networks—specifically the rapid suppression of auxin (IAA) and the immediate accumulation of stress-responsive hormones (ABA, JA)—suggest an active evolutionary strategy. The rootstock actively ceases normal root expansion to redirect extensive energy resources toward defense signaling. Furthermore, the robust, early-stage upregulation of the GSH-GST and HSP networks highlights that neutralizing ROS bursts and preventing severe protein misfolding are the primary, immediate-early defense mechanisms executed by the rootstock to counteract heavy metal toxicity long before macroscopic tissue necrosis becomes visible. Cd²^+^ treatment significantly disrupted hormone homeostasis in the roots, consistent with the large number of DEGs enriched in hormone-related pathways observed in the transcriptomic analysis. Phytohormones, present in varying concentrations, act as chemical messengers in plant cells (Voß et al., 2014). Plants face many environmental stresses, and understanding how they perceive and respond to these stresses, like excess HMs, is crucial (Bücker-Neto et al., 2017). Hormones help link plants to environmental signals, regulating growth and development, especially under stress (Verslues, 2016). In addition, a significant upregulation of genes associated with GSH, GST, GRX, CAT, and APR were identified following Cd²^+^ treatment. Previous studies have demonstrated that Cd²^+^ often induces the generation of toxic ROS, including hydrogen peroxide, hydroxyl radicals, and superoxide anions (Huihui et al., 2020). These ROS can oxidize biological macromolecules such as lipids, proteins, and nucleic acids, leading to enzyme inactivation, lipid peroxidation, and membrane damage. To counteract ROS-induced damage, plants have evolved antioxidant defense mechanisms, including enzymatic antioxidants like CAT, SOD, and GSH (Maleki et al., 2017). GSH plays a dual role as an antioxidant in mitigating redox imbalance caused by heavy metal accumulation and as a precursor for phytochelatins, which are critical for Cd²^+^ chelation (Hossain et al., 2012; Asgher et al., 2017). GSH also serves as a substrate for GSTs, which catalyze the conjugation of GSH with xenobiotics and the reduction of toxic organic hydroperoxides (Kumar and Trivedi, 2018; Wu et al., 2019). GRXs, small redox proteins that utilize GSH as a cofactor, are similarly induced by heavy metals (Wu et al., 2017). The synthesis of GSH in plants relies on sulfur assimilation, beginning with sulfate (SO_4_²^-^;) uptake and its conversion to adenosine 5′-phosphosulfate (APS) by APR, followed by reduction to sulfite (SO_3_²^-^;) and sulfide (S²^-^;), which are utilized for cysteine and GSH production (Hernández et al., 2015). In Arabidopsis, APR2 positively regulates Cd tolerance through glutathione-dependent pathway (Xu et al., 2020). The upregulation of GSH, GST, GRX, CAT, and APR-related genes under Cd²^+^ treatment suggests that Cd²^+^ may induce ROS production in kiwifruit roots. Additionally, HSPs, which are expressed in response to stressors and serve as bio-indicators of oxidative stress, were significantly upregulated after Cd²^+^ treatment. This further indirectly supports the hypothesis that Cd²^+^ induces ROS generation in kiwifruit cells, while also increasing protein misfolding or damage. Together, these findings highlight the critical role of antioxidant and stress-responsive mechanisms in mitigating Cd²^+^-induced oxidative stress in *A. valvata*.

## 5 Conclusions

Our study reveals that the kiwifruit rootstock *A. valvata* exhibits outstanding traits, including resistance to canker disease, waterlogging, and Al³^+^ stress, as well as a high survival rate in cutting propagation. We also developed an efficient root genetic transformation system for *A. valvata* and *M. rubra* that bypassing tissue culture. Using GFP fluorescence intensity as a marker, we demonstrated that Cd can significantly impact the physiological state of kiwifruit cells within 2 hours. Furthermore, we analyzed the effects of Cd treatment on whole-genome gene transcription levels and hormone content in *A. valvata*.

## Data Availability

The datasets presented in this study can be found in online repositories. The names of the repository/repositories and accession number(s) can be found in the article/**Supplementary Material**.
