# Supplementary figures and images for: Integrated transcriptomic and physiological analysis reveals cadmium stress responses in kiwifruit rootstock Actinidia valvata via an optimized Agrobacterium rhizogenes-mediated hairy root transformation system
Source: Front Plant Sci. 2026 Apr 28;17:1818881. doi: 10.3389/fpls.2026.1818881 (PMC13160912; doi:10.3389/fpls.2026.1818881)

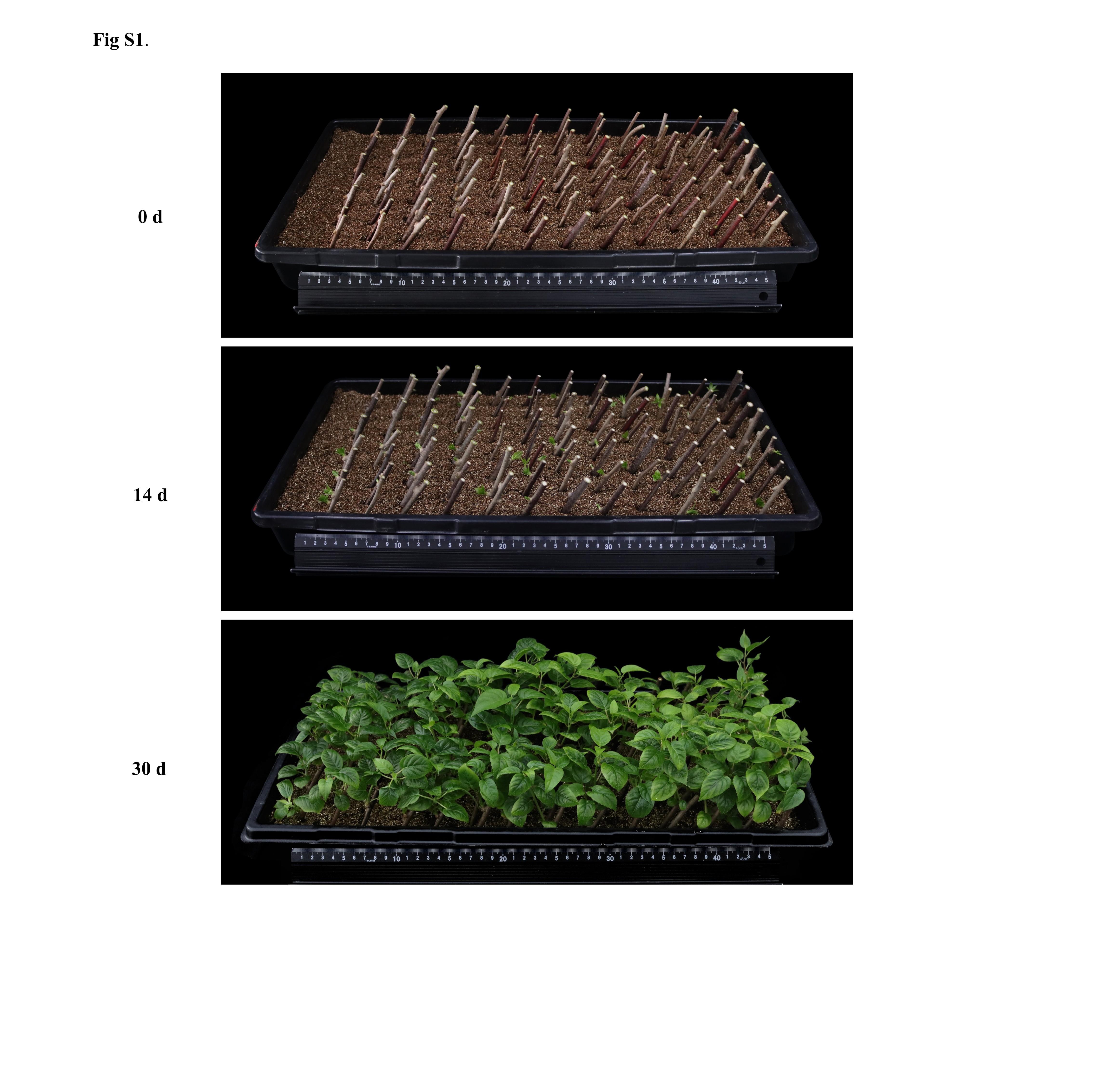

Supplement: Supplementary file 1 [file Image1.tiff]

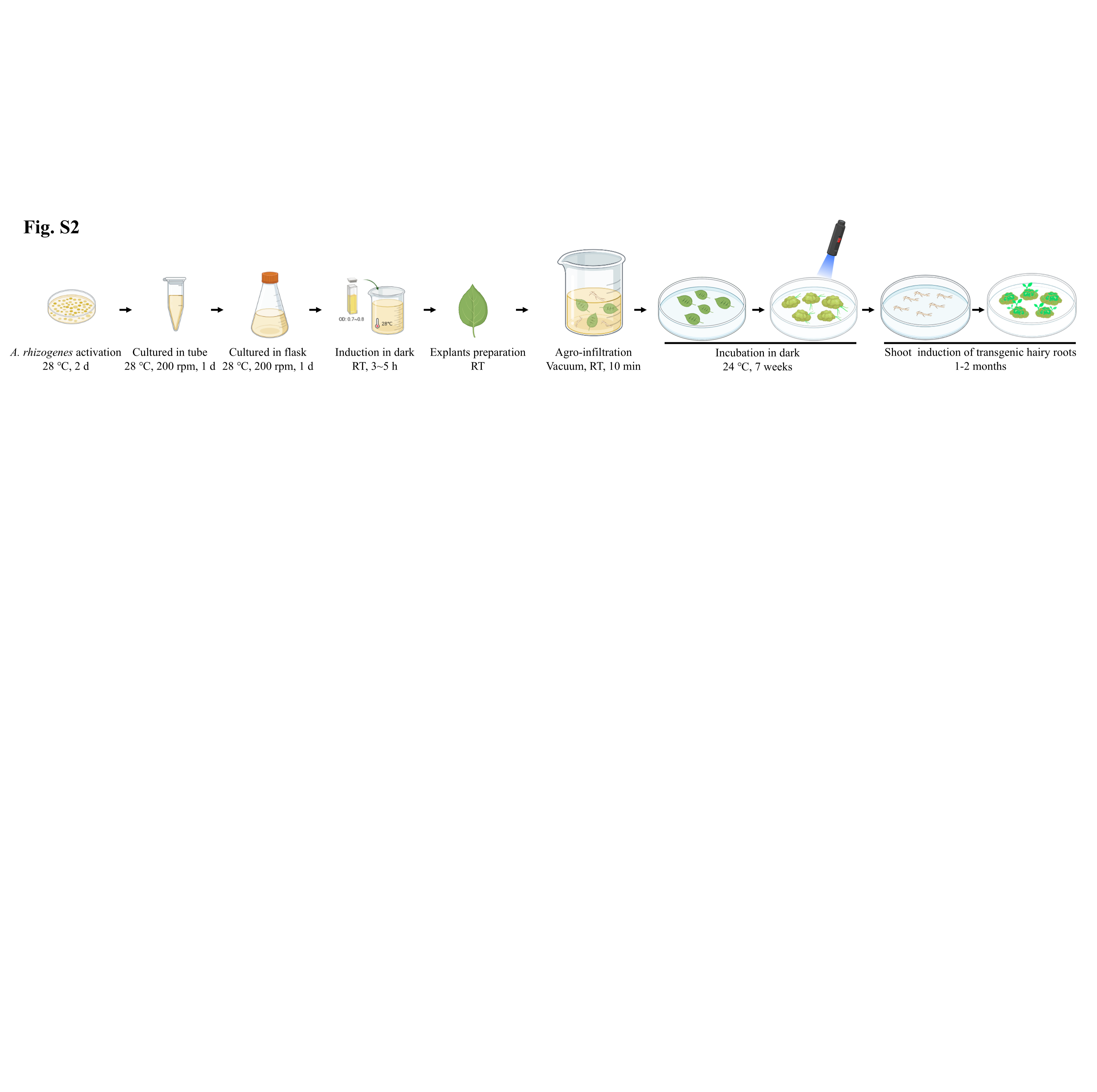

Supplement: Supplementary file 2 [file Image2.tiff]

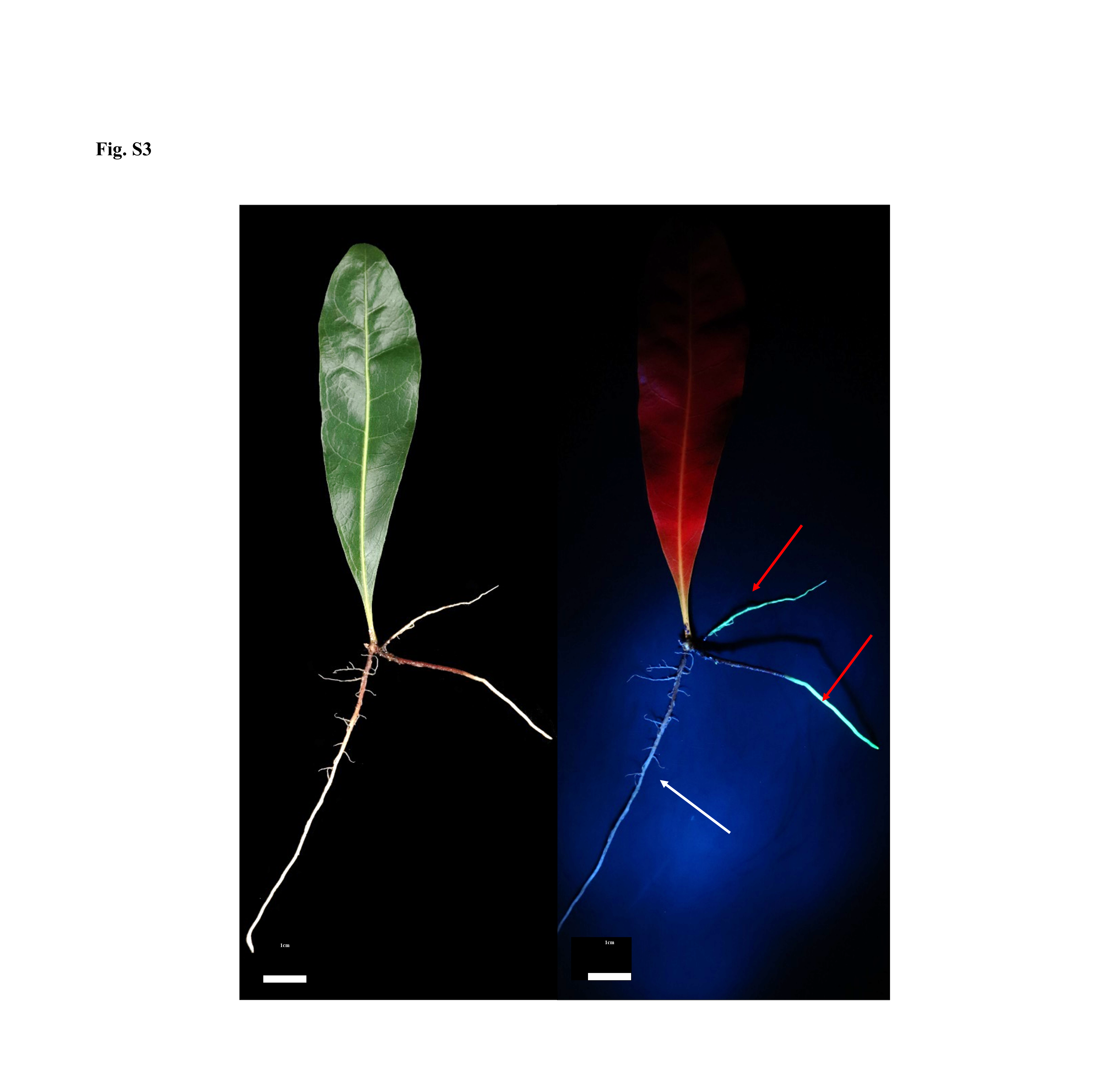

Supplement: Supplementary file 3 [file Image3.tiff]

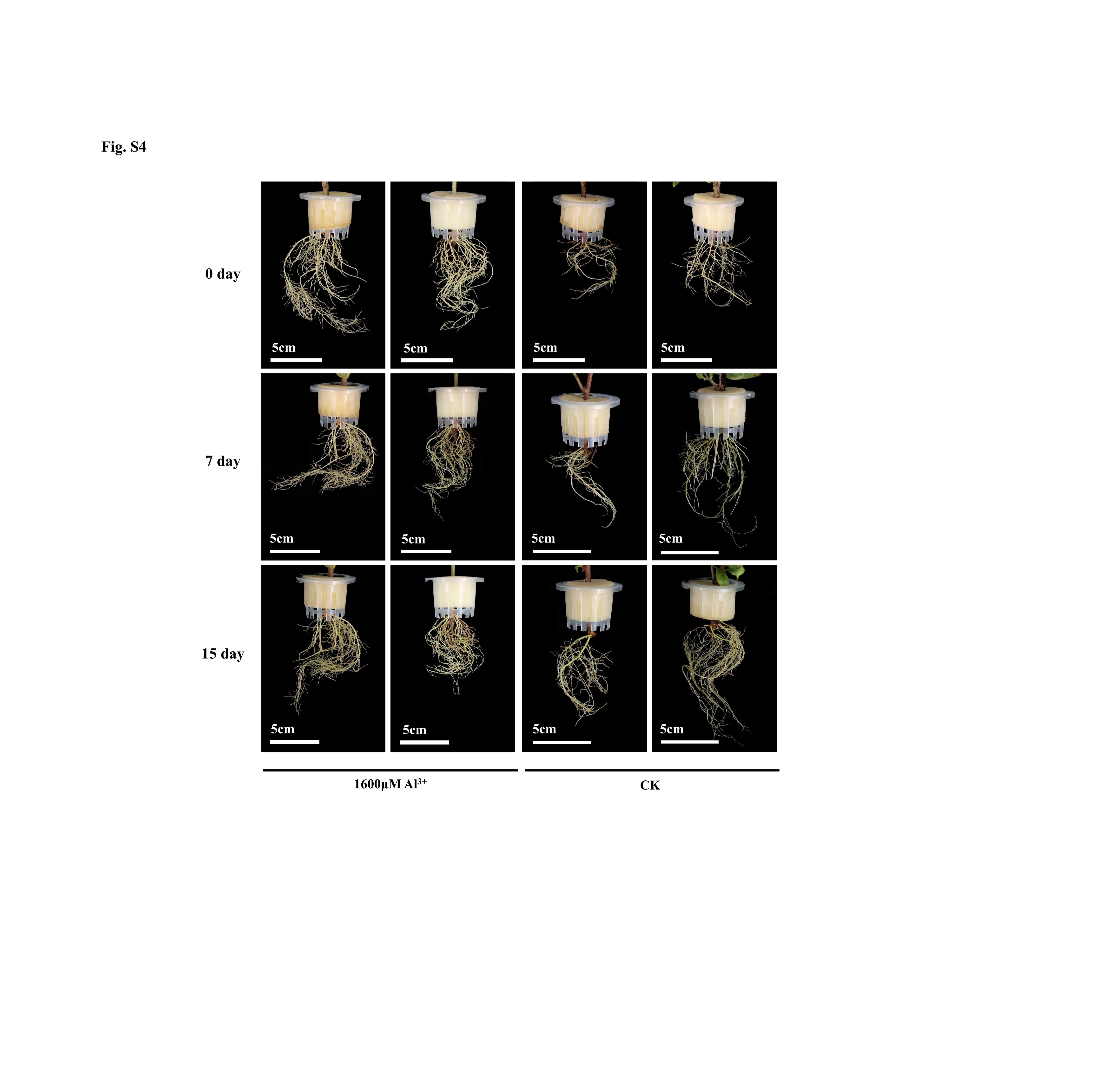

Supplement: Supplementary file 4 [file Image4.tiff]
